# A Branched and Double Alpha-Gal-Bearing Synthetic Neoglycoprotein as a Biomarker for Chagas Disease

**DOI:** 10.3390/molecules27175714

**Published:** 2022-09-05

**Authors:** Alba L. Montoya, Elisa G. Carvajal, Uriel Ortega-Rodriguez, Igor L. Estevao, Roger A. Ashmus, Sohan R. Jankuru, Susana Portillo, Cameron C. Ellis, Colin D. Knight, Julio Alonso-Padilla, Luis Izquierdo, Maria-Jesus Pinazo, Joaquim Gascon, Veronica Suarez, Douglas M. Watts, Iliana R. Malo, Janine M. Ramsey, Belkisyolé Alarcón De Noya, Oscar Noya, Igor C. Almeida, Katja Michael

**Affiliations:** 1 Department of Chemistry and Biochemistry, Border Biochemical Research Center, The University of Texas at El Paso, El Paso, TX 79968, USA; 2 Department of Biological Sciences, Border Biochemical Research Center, The University of Texas at El Paso, El Paso, TX 79968, USA; 3 Barcelona Institute for Global Health (ISGlobal), 08003 Barcelona, Spain; 4 Consorcio Centro de Investigación Biomédica en Red (CIBER) de Enfermedades Infecciosas, Instituto de Salud Carlos III (CIBERINFEC, ISCIII), 28029 Madrid, Spain; 5 Hospital Clínic de Barcelona, 08036 Barcelona, Spain; 6 Centro Regional de Investigación en Salud Pública, Instituto Nacional de Salud Pública, Tapachula 30700, Chiapas, Mexico; 7 Sección de Inmunología, Instituto de Medicina Tropical, Universidad Central de Venezuela, Caracas 1041, Venezuela; 8 Seccion de Biohelmintiasis, Instituto de Medicina Tropical, Universidad Central de Venezuela, Caracas 1041, Venezuela

**Keywords:** Chagas disease, *Trypanosoma cruzi*, anti-α-Gal antibodies, biomarker, α-Gal-containing neoglycoprotein, chemotherapy, oligosaccharide synthesis

## Abstract

Chagas disease (CD) is caused by the parasite *Trypanosoma cruzi* and affects 6–7 million people worldwide. The diagnosis is still challenging, due to extensive parasite diversity encompassing seven genotypes (TcI-VI and Tcbat) with diverse ecoepidemiological, biological, and pathological traits. Chemotherapeutic intervention is usually effective but associated with severe adverse events. The development of safer, more effective therapies is hampered by the lack of biomarker(s) (BMKs) for the early assessment of therapeutic outcomes. The mammal-dwelling trypomastigote parasite stage expresses glycosylphosphatidylinositol-anchored mucins (tGPI-MUC), whose *O*-glycans are mostly branched with terminal, nonreducing α-galactopyranosyl (α-Gal) glycotopes. These are absent in humans, and thus highly immunogenic and inducers of specific CD anti-α-Gal antibodies. In search for α-Gal-based BMKs, here we describe the synthesis of neoglycoprotein NGP11b, comprised of a carrier protein decorated with the branched trisaccharide Galα(1,2)[Galα(1,6)]Galβ. By chemiluminescent immunoassay using sera/plasma from chronic CD (CCD) patients from Venezuela and Mexico and healthy controls, NGP11b exhibited sensitivity and specificity similar to that of tGPI-MUC from genotype TcI, predominant in those countries. Preliminary evaluation of CCD patients subjected to chemotherapy showed a significant reduction in anti-α-Gal antibody reactivity to NGP11b. Our data indicated that NGP11b is a potential BMK for diagnosis and treatment assessment in CCD patients.

## 1. Introduction

American trypanosomiasis, commonly known as Chagas disease (CD), is a neglected tropical disease (NTD) caused by the protozoan parasite *Trypanosoma cruzi*. Historically limited to Latin America, CD has spread beyond its geographical boundaries due to global migratory flows and currently affects 6–7 million people worldwide [1,2,3,4,5]. Two studies have estimated that approximately 238,000 or 300,000 individuals are chronically infected with *T. cruzi* in the United States, posing a serious threat of transmission [6,7]. *T. cruzi* transmission occurs by several routes, including insect-vector transmission by Triatominae species (commonly known as kissing bugs) and other non-vectorial mechanisms, which include blood transfusion, organ transplantation, consumption of contaminated foods and juices, and vertical transmission from mother to infant [2,8,9,10].

Clinical manifestations of CD can be broken down into two distinct phases, i.e., acute and chronic. The acute phase is usually characterized by nonspecific symptoms, which include fever and malaise. However, acute myocarditis and meningoencephalitis may occur in 5–10% of patients. In contrast, the chronic phase is characterized by a broad spectrum of clinical outcomes, ranging from complete lack of symptoms to severe disease or even death [8,11]. An estimated 20–30% of individuals with chronic CD (CCD) will develop cardiomyopathy, leading to cardiac failure and sudden death. In contrast, others can experience the development of gastrointestinal complications, including megacolon or megaesophagus [4,5,8,12].

The diagnosis and treatment of CD can be problematic, owing to the existence of seven discrete typing units (DTUs) or genotypes (TcI-TcVI and Tcbat) with highly diverse ecoepidemiological, geographical, biological, and pathological traits, which result in several different strains that cause different clinical manifestations [13,14]. Currently, only two drugs are available for the chemotherapy of CD, i.e., nifurtimox (NFX) and benznidazole (BNZ). While these drugs are both highly (90–100%) effective in the acute phase, they are moderately (60–80%) effective in the chronic phase [2,15]. Moreover, both drugs cause severe adverse events, resulting in 20–30% of patients permanently interrupting the treatment [15,16]. Furthermore, patients take approximately 10–20 years to exhibit negative seroconversion with conventional serology (CS), making the assessment of chemotherapeutic efficacy mostly infeasible in clinical settings. In clinical trials, polymerase chain reaction (PCR) has been used as a gold standard to detect therapeutic failure. However, since the number of circulating parasites in chronic patients is usually low and even more so in treated patients, PCR cannot be used as a measure to detect cure or parasite elimination. PCR is only valuable when it is positive, indicating therapeutic failure. The lack of specific clinical biomarkers (BMKs) for CD chemotherapy poses a major burden for developing and evaluating novel anti-trypanosomal drugs [17]. Poor prognostic perspectives from CS do not support widespread treatment of chronic CD, resulting in only 1% of patients undergoing treatment [9,18].

*T. cruzi* has a highly complex cell surface that harbors various types of glycoconjugates linked to the plasma membrane through a glycosylphosphatidylinositol (GPI) anchor. They include major families of GPI-anchored mucins (TcMUC or GPI-MUC), mucin-associated surface proteins, *trans*-sialidases, and protein-free glycoinositolphospholipids (GIPLs) [19,20,21,22]. GPI-MUC are the most abundant glycoconjugates decorating the parasite surface, consisting of 2 × 10^6^ copies per parasite and expressed by hundreds of genes [21,23]. Approximately 60% of the *O*-glycans found on the GPI-MUC from the infective, mammal-dwelling trypomastigote stage (tGPI-MUC) contain terminal, nonreducing α-galactopyranosyl (α-Gal) residues. Only one tGPI-MUC *O*-glycan has been fully characterized to date, i.e., the linear trisaccharide, Galα1,3Galβ1,4GlcNAcα, which makes up ~10% of all *O*-glycans in tGPI-MUC [24]. Most of the remaining 90% of *O-*glycans are believed to be branched at the reducing α-GlcNAc terminus; however, their exact structures remain uncharacterized [24]. These α-Gal-containing glycans or glycotopes are highly immunogenic to humans, due to their absence on human glycoproteins and glycolipids as a result of the inactivation of the α1,3-galactosyltransferase (α1,3-GalT) gene millions of years ago in our primate ancestors [25,26]. Patients with CD produce substantial amounts of lytic, protective anti-α-Gal antibodies (Abs) against α-Gal glycotopes expressed on tGPI-MUC. These CD anti-α-Gal Abs are the major protective antibodies in both acute and chronic phases of the disease [24,27,28,29,30], and are present in the sera of CCD patients from different endemic (e.g., Brazil, Argentina, Chile, Bolivia, Mexico, Venezuela) and nonendemic countries (e.g., Spain, United States), signifying that these epitopes are universally expressed in different strains and genotypes of *T. cruzi* [31,32,33,34,35,36,37]. Unlike protein-specific antibodies, glycan-specific antibodies disappear from circulation soon, i.e., within a few years, after the elimination of the parasite from the infected host [32,37,38], suggesting that α-Gal-containing glycotopes of tGPI-MUC may not only be highly specific BMKs for accurate diagnosis but could also be instrumental in the early assessment of postchemotherapeutic outcomes, as previously demonstrated [31,32,33,35,37,38,39]. In fact, tGPI-MUC (a.k.a., F2/3 or AT antigen), purified from the Y strain, was the first BMK to be validated in a clinical trial for early assessment of cure in adolescents with early CCD [37,38]. Synthetic neoglycoproteins (NGPs) based on the terminal nonreducing α-Gal residues found in the tGPI-MUC [39,40,41] could thus be a promising diagnostic tool for CCD diagnosis and prognosis of therapeutic outcomes, providing a more rapid and accurate test and avoiding very demanding long-term patient follow-up. The serological probing of NGPs decorated with the terminal glycostructures of known or partially known parasite-derived glycans can be regarded as reversed immunoglycomics. In this approach, information about the antigenicity of synthetic homogeneous oligosaccharides is obtained, rather than relying on the cultivation of parasites and the isolation and study of their glycoconjugates. Similar reversed immunoglycomic approaches have recently led to the discovery of β-galactofuranose (β-Gal*f*)-based BMKs for CCD diagnosis [42], and α-Gal-containing BMKs for the diagnosis of Old-World and New-World (American) cutaneous or tegumentary leishmaniases [43,44].

Here, we describe the synthesis of neoglycoprotein NGP11b, the assessment of its suitability as a BMK for CCD diagnosis, and its potential for the monitoring of disease clearance after chemotherapy. NGP11b consists of bovine serum albumin (BSA) with multiple copies of the putative tGPI-MUC-derived glycotope Galα1,2[Galα1,6]Galβ covalently linked via 4-(N-succinimidomethyl)cyclohexane-1-carbonyl linkers. The oligosaccharide contains a 2,6-branched Gal unit present in some of the tGPI-MUC *O*-glycans, as previously revealed by methylation and hydrolysis experiments [24]. The capability of NGP11b to serve as a diagnostic BMK in patients was assessed by chemiluminescent enzyme-linked immunosorbent assay (CL-ELISA), using sera of CCD patients from Venezuela and Mexico. The antibody reactivities of *T. cruzi*-positive sera and negative controls to NGP11b and purified tGPI-MUC from the Colombiana (DTU TcI) and Y (DTU TcII) strains [13,45] were compared. In addition, we describe a preliminary assessment of NGP11b as a BMK in the monitoring of anti-α-Gal antibody levels in patients who had undergone treatment with benznidazole (BZN).

## 2. Results and Discussion

### 2.1. Chemical Synthesis of NGP11b

The synthesis of 3-thiopropyl glycoside of Galα1,2[Galα1,6]Galβ (**G11_SH_**) with two terminal nonreducing α-Gal moieties and its conjugation with commercially available maleimide-derivatized BSA is presented. Structural analysis of tGPI-MUC *O-*glycans of *T. cruzi* (Y strain, DTU TcII) [24] and preliminary interrogations of an α-Gal-containing glycoarray by CL-ELISA using sera from CCD patients suggested that this branched glycan could be a terminal partial structure of *O-*glycans of tGPI-MUC [40,46].

**Figure 1:**
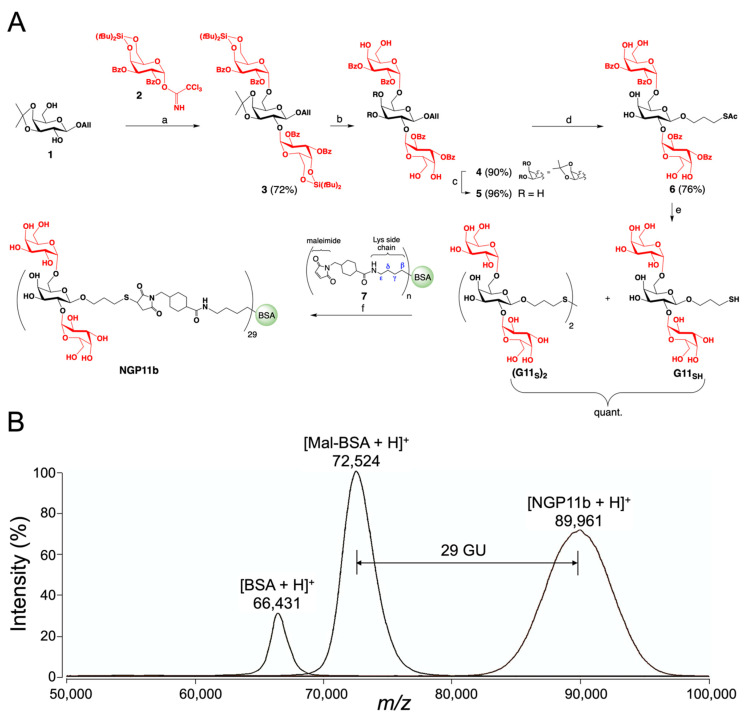
(**A**) Synthesis of the trisaccharide 3-thiopropyl α-D-galactosyl-(1,2)-[α-D-galactosyl-(1,6)]-β-D-galactoside (**G11_SH_**) and its conjugation with maleimide-derivatized BSA to afford NGP11b. (a) TMSOTf, DCM; (b) HF-pyr, THF; (c) DCM, TFA, H_2_O; (d) AcSH, AIBN, THF, UV light (350 nm); (e) NaOMe, MeOH; (f) TCEP-HCl, pH 7.2. (**B**) Overlaid MALDI-TOF mass spectra of singly charged molecular ions of BSA ([BSA + H]^+^), maleimide-BSA ([Mal-BSA + H]^+^), and NGP11b ([NGP11b + H]^+^) are indicated. GU, glycan unit; *m/z*, mass to charge ratio.

NGP11b was prepared by the synthetic strategy depicted in Figure 1A, and consisted of the following steps: (a) a double glycosylation of the partially protected acceptor **1** using the stereoselective introduction of α-Gal moieties with Kiso’s α-Gal donor **2** equipped with a 4,6-*O*-di-*tert*-butyl silylene group [47]; (b) trimethylsilyl trifluoromethanesulfonate (TMSOTf) activation to furnish the fully protected trisaccharide **3** in 72% yield; (c) the removal of the silylene group using hydrofluoric acid–pyridine (HF-pyr) complex in 90% yield; (d) hydrolysis of the isopropylidene group with TFA/H_2_O/DCM in 96% yield; (e) the installation of a 3-thiopropyl group at the reducing end of the glycan by radical addition of thioacetic acid (AcSH) to the allyl glycoside **5** in the presence of azobisisobutyronitrile (AIBN) in anhydrous THF under UV-light illumination affording thioester **6** in 76% yield; (f) the complete deacylation of thioester **6** using Zemplén conditions affording the target trisaccharide **G11_SH_**, which oxidized on air to the disulfide **(G11_S_)_2_** within approximately 1 h; and (g) the conjugation of glycan **G11_SH_** obtained by reduction of disulfide **(G11_S_)_2_** with tris(2-carboxyethyl)phosphine hydrochloride) (TCEP^-^HCl) in situ with commercial maleimide-derivatized bovine serum albumin (BSA) (**7**). The intermediates **3**–**6** and the disulfide **(G11_S_)_2_** were fully characterized by thin-layer chromatography (TLC), optical rotation, ^1^H and ^13^C nuclear magnetic resonance (NMR) spectroscopy, and high-resolution electrospray ionization–time-of-flight mass spectrometry (HR ESI-TOF-MS). The average number of conjugated glycan units (GU) per BSA molecule, i.e., Galα(1,2)[Galα(1,6)]Galβ units/BSA, was determined by matrix-assisted laser desorption/ionization–time-of-flight mass spectrometry (MALDI-TOF-MS) (Figure 1B). Subtracting the average molecular mass of the commercial maleimide-derivatized BSA from the average mass of NGP11b and dividing by the molecular mass of the glycan plus linker gave an average of 29 glycan units (GU) per BSA molecule (Figure 1B).

### 2.2. Evaluation of NGP11b as a Biomarker for Chagas Disease by CL-ELISA

**Figure 2:**
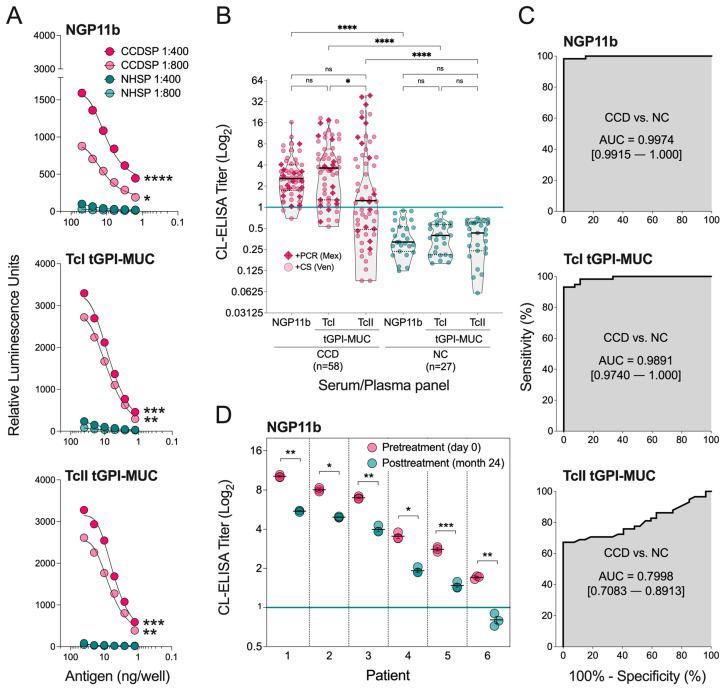
Serological evaluation of NGP11b, TcI tGPI-MUC, and TcII tGPI-MUC by CL-ELISA. (**A**) Cross-titration of varying concentrations (50, 25, 12.5, 6.2, 3.1, and 1.6 ng/well) of synthetic NGP11b (top panel), and purified TcI tGPI-MUC (from Colombiana strain, 40-2 ng/well) (middle panel) and TcII tGPI-MUC (from Y strain, 40–2.5 ng/well) (bottom panel) at 1:400 and 1:800 serum dilutions. The CCD serum pool (CCDSP) (*n* = 10) was obtained from CCD patients from Venezuela. The normal human serum pool (NHSP) (*n* = 10) was obtained from healthy donors from the U.S.A. (**B**) CL-ELISA titers of sera (at 1:800 dilution) of individual CCD patients (*n* = 58), from Venezuela (*n* = 42) and Mexico (*n* = 16), and umbilical cord plasma (at 1:400 dilution) samples of healthy individuals, negative control (NC) (*n* = 27) from the U.S.A. to NGP11b, TcI tGPI-MUC, and TcII tGPI-MUC. Serum samples from Mexico (Mex) with a confirmed CD diagnosis by positive PCR (+PCR), and from Venezuela (Ven) with a confirmed CD diagnosis by positive conventional serology (+CS), are indicated by different symbols. Green line, cutoff value (CL-ELISA titer = 1.000), calculated as described in Materials and Methods. (**C**) ROC curves for NGP11b, TcI tGPI-MUC, and TcII tGPI-MUC comparing the reactivity of sera from CCD patients vs. healthy individuals, using the data depicted in the truncated violin scatterplot (**B**). Statistical analysis: Kruskal–Wallis test with Dunn’s multiple comparison test with Geisser–Greenhouse correction. * *p* < 0.05; ** *p* < 0.01; *** *p* < 0.001; **** *p* < 0.0001. The area under the curve (AUC) (gray area) and its value are indicated. The 95% confidence interval (CI) values are shown in brackets. (**D**) CL-ELISA titers of CCD patients before treatment (day 0) and after treatment (24 months) with the standard of care of benznidazole (150 mg twice a day, for 60 days). Statistical analysis: mixed-effects model with Geisser–Greenhouse correction. * *p* < 0.05; ** *p* < 0.01; *** *p* < 0.001.

With NGP11b in hand, its suitability as a diagnostic BMK was assessed and compared with purified tGPI-MUC from the mammal-dwelling trypomastigote stage of *T. cruzi* Colombiana (TcI tGPI-MUC) and Y (TcII tGPI-MUC) strains, as described in Materials and Methods. We used tGPI-MUC preparations from these two genotypes because they are responsible for most *T. cruzi* infections in North, Central, and South America and Spain [14]. Moreover, since most of the CCD serum samples used in this study were from Venezuela and Mexico, we evaluated whether there was any difference in the immunoreactivity to NGP11b (29 GU/BSA) in comparison with that to TcI tGPI-MUC and TcII tGPI-MUC. To that end, NGP11b and the purified tGPI-MUC preparations were immobilized in 96-well microplates, and antibody-binding responses were measured by CL-ELISA using pooled sera from CCD patients (*n* = 10, CCDSP) from Venezuela, and non-CD, healthy individuals (*n* = 10, NHSP) from the U.S.A. To optimize the CL-ELISA conditions for maximum specificity and sensitivity, a cross-titration with two dilutions (1:400 and 1:800) of pooled sera and different quantities of immobilized antigens was performed. As shown in Figure 2A, the synthetic NGP11b and both purified TcI and TcII tGPI-MUC preparations exhibited significant differential immunoreactivity between CCDSP and NHSP. The reactivity of NHSP was significantly lower than CCDSP at both serum dilutions and various antigen concentrations, indicating that natural (or normal human serum, NHS) anti-α-Gal Abs from healthy individuals, present in all humans [25], reacted weakly with NGP11b and both TcI and TcII tGPI-MUC preparations. In contrast, *T. cruzi-*specific anti-α-Gal Abs from CCD patients exhibited significantly higher reactivities in both serum dilutions in an antigen concentration-dependent manner. The much lower reactivity of NHS anti-α-Gal Abs than anti-α-Gal Abs from CD patients (Ch anti-α-Gal Abs) to purified tGPI-MUC from the Y strain (TcII) was initially demonstrated by Almeida et al. [24]. Moreover, the fact that most (~90%) α-Gal-bearing *O*-glycans are branched, as discussed above, the stronger reactivity of CCDSP, and consequently the Ch anti-α-Gal Abs to NGP11b may indicate that this branched glycotope could be immunodominant in TcI tGPI-MUC. In a previous comparative serological study with pooled sera of CCD patients, an NGP antigen similar to NGP11b was included [40]. It differed from NGP11b in the linker that connected Galα1,2[Galα1,6]Galβ units to BSA, and possibly also in the average number of GU/BSA, which had not been determined. However, since control experiments showed negligible binding to the linker [40], the lower Ab reactivity and the smaller differential between CCD patient sera vs. NHS previously observed is more likely attributable to different assay parameters used, i.e., an unoptimized serum dilution and antigen quantity, and possibly to the fact that CCD patient sera were from donors originally from Bolivia [40], where a different *T. cruzi* DTU, i.e., genotype TcV is prevalent [13].

The ability of NGP11b to accurately differentiate sera/plasma of individual CCD patients from that of healthy individuals is essential for its usefulness as a diagnostic BMK. To further assess the sensitivity, specificity, and other parameters of NGP11b as a diagnostic BMK for CCD, antibody-binding responses were determined using 58 sera of patients with CCD (from Mexico and Venezuela) and 27 umbilical cord plasma samples from healthy (non-CCD) individuals by CL-ELISA, and by comparing with tGPI-MUC (Figure 2B). When NGP11b was used as an antigen in CL-ELISA, CCD sera and healthy plasma controls fell into two distinct groups, with little overlap between the two categories, and only 3/58 (5.2%) sera were diagnosed as false-negative (Table 1, Figure 2B). A similar trend to NGP11b was observed for TcI tGPI-MUC, but with a higher number (*n* = 7, 12.1%) of false-negative results. In contrast, TcII tGPI-MUC exhibited a very large number (*n* = 26, 44.8%) of false-negative results (Table 1, Figure 2B). When sera of CCD patients from Mexico, who had CD diagnosis confirmed by PCR (dark pink diamond plus, *n* = 16), were evaluated, we observed that 100% (16/16), ~88% (14/16), and ~69% (11/16) were positive for NGP11b, TcI tGPI-MUC, and TcII tGPI-MUC, respectively (Figure 2B). This indicates a similar overall reactivity of these selected sera from Mexico when compared with the whole serum panel (Table 1). Likewise, CCD sera of Venezuela (light pink circles, *n* = 42), which were diagnosed only by CS, showed a comparable trend, that is, ~93% (39/42), ~88% (37/42), and ~52% (22/42) were positive for NGP11b, TcI tGPI-MUC, and TcII tGPI-MUC, respectively (Figure 2B). Since most patients of the Mexico and Venezuela panel were likely infected with TcI, which is a highly prevalent genotype infecting people in these countries [14], these results indicate that NGP11b could be a very promising diagnostic BMK to be used in countries where the highly diverse TcI genotype is predominant.

Considerable variation in the immunological and biological properties and expression of the terminal, nonreducing α-Gal glycotopes have previously been observed in tGPI-MUC purified from Colombiana (TcI) and Y (TcII) strains [14,48]. In this study, we observed a similar immunoreactivity (no significant difference) to NGP11b and TcI tGPI-MUC (from Colombiana strain) of sera from CCD patients (Figure 2B), indicating that the Galα1,2[Galα1,6]Galβ glycotope is likely immunodominant in TcI tGPI-MUC (from Colombiana strain) and less abundant in TcII tGPI-MUC (from Y strain). However, it remains to be confirmed whether tGPI-MUCs from other strains belonging to DTU TcI, prevalent in Northern South America (e.g., Venezuela and Colombia) and Central and North America [14] would behave similarly regarding seroreactivity in comparison to tGPI-MUCs from other TcII strains. Obviously, an in-depth structural and quantitative analysis of α-Gal-containing *O*-glycans of tGPI-MUC from both strains and other TcI and TcII strains should be performed in due course.

Receiver-operating characteristic (ROC) curves were plotted to compare the sensitivity and specificity of NGP11b, TcI tGPI-MUC, and TcII tGPI-MUC (Figure 2C). The area under the curve (AUC) values obtained from the ROC curves indicate that NGP11b (AUC = 0.9974) exhibited higher sensitivity and specificity than TcI tGPI-MUC (AUC = 0.9891) and TcII tGPI-MUC (AUC = 0.7998). The specificity of all three antigens was 100% (Table 2), indicating that all of them can accurately rule out CCD caused by the Colombiana or Y strain, or related strains, expressing a glycotope identical or similar to that of NGP11b. On the other hand, the sensitivity was variable within the three antigens. Using a titer cutoff value of 1.000, TcI tGPI-MUC, TcII tGPI-MUC, and NGP11b had sensitivities of 87.9%, 55.2%, and 94.8%, respectively (Table 2). These data demonstrate that NGP11b has potential as a diagnostic serological BMK.

How can the higher sensitivity of NGP11b compared to TcI and TcII tGPI-MUC be explained? Without a detailed analysis of the *O-*glycans of these purified *T. cruzi* glycoproteins, and assuming that both TcI and TcII tGPI-MUC exhibit Galα1,2[Galα1,6]Galβ or a similar glycotope, one could hypothesize that NGP11b (with 29 GU/BSA) has a higher density of that glycan than TcI and TcII tGPI-MUC. Glycan density has been a critical factor for modulating the binding of anti-glycan antibodies or lectins in different types of ELISA- or glycan array-based immunoassays, as previously demonstrated [49,50,51]. For instance, Oyelaram et al. [50] showed that a variable carbohydrate density of the tumor-associated Tn antigen (GalNAcα-*O*-), Tn-based (e.g., clustered Tn [Tn3], S-Tn(Ser)-S, S-Tn(Thr)-S), and Tn-unrelated glycan structures, covalently linked to BSA as NGPs on a microarray, could significantly influence the apparent binding (or association/affinity) and dissociation constants of lectins, monoclonal antibodies, and human serum antibodies. Moreover, the evaluation of these neoglycoproteins at distinct glycan densities resulted in the detection of different serum antibody subpopulations among individuals, which could not be detected using a single glycan density. Chiodo et al. [49] showed that a higher glycan density could lead to increased sensitivity in the detection of anti-glycan antibodies against HIV gp120 and *Streptococcus pneumoniae* capsular polysaccharide by gold glyconanoparticle (GNP) ELISA. We are currently evaluating the influence of the glycan density on NGP11b and several other α-Gal-bearing NGPs in the recognition by anti-glycan antibodies in the context of CD to further improve the sensitivity of the CL-ELISA using these NGPs.

While NGP11b holds promise as a BMK for CD diagnosis, another important question is whether or not it is also suitable for the assessment of chemotherapy efficacy in patients. To address this question, the anti-α-Gal antibody levels of six CCD patients who were treated in Barcelona, Spain were determined before and after treatment with BNZ using NGP11b as a BMK in CL-ELISA. Our data showed that even though individual patients had different anti-α-Gal antibody levels prior to chemotherapy, 24 months after chemotherapy, all patients showed a statistically significant reduction in anti-α-Gal antibody levels (Figure 2D). On average, a drop of 45% of the CL-ELISA titers 24 months posttreatment was observed, indicating that NGP11b can follow α-Gal-specific antibodies that begin to disappear from circulation concurrently or soon after the elimination of *T. cruzi* from the host, as previously observed for tGPI-MUC in treated children and adolescents with early CCD [37,38]. Of course, this preliminary observation with NGP11b needs to be expanded with a larger cohort of treated patients to validate this NGP to be used in clinical settings. To this end, our ongoing phase II clinical trial in Bolivia, named TESEO (ClinicalTrials.gov identifier NCT03981523), will evaluate NGP11b and several other α-Gal-bearing NGPs in the coming years as BMK(s) for early assessment of therapeutic outcomes of CCD [52].

Taken together, our data suggested that NGP11b can be utilized as a CCD-specific BMK for diagnosis and drug efficacy assessment. NGP11b may eventually replace tGPI-MUC, which lacks specificity, batch-to-batch consistencies, and requires the cultivation of a large number of infectious trypomastigote forms, followed by a lengthy purification process.

## 3. Materials and Methods

### 3.1. General Information

All chemicals were purchased as reagent grade and used without further purification from Thermo Fisher Scientific, Sigma-Aldrich, or Acros Organic, USA. The ACS grade solvents used for reactions were obtained from Thermo Fisher Scientific, and if necessary, further dried following standard procedures. Molecular sieves (MS 3 and 4 Å) were purchased from Alfa Aesar or Fisher Scientific, respectively, and activated under high vacuum and heat prior to use. Reactions were performed under an argon (Ar) atmosphere, under strictly anhydrous conditions, and monitored by thin-layer chromatography (TLC) on silica gel 60 F254 plates from EMD Millipore or Dynamic Adsorbents, Inc. Spots were detected under UV light and/or by charring with 2% sulfuric acid in ethanol. Compound purifications were performed by column chromatography on silica gel (40–60 μm) from Fisher Chemical, and the ratio between silica and crude product ranged from 50:1 to 200:1 (dry *w*/*w*). Optical rotation measurements were obtained on an ATAGO AP300 automatic polarimeter. ^1^H and ^13^C NMR spectra were recorded on a Bruker Avance III HD 400 MHz NMR spectrometer at 400 and 101 MHz, respectively. Chemical shifts (in ppm) were determined relative to tetramethylsilane (δ 0.00 ppm). Coupling constant(s) [Hz] were measured from one-dimensional spectra. Mass spectrometry (MS) of the carbohydrate derivatives was performed on a high-resolution JEOL Accu Time-of-Flight (TOF) mass spectrometer using an electrospray ionization (ESI) source. Protein derivatives were measured by MALDI-TOF-MS (MALDI-8020, Shimadzu). The thiol-ene reaction was performed in a Rayonet RPR200 photochemical reactor (Southern New England Ultraviolet, Branford, CT) equipped with 16 UV lamps, at 350 nm.

### 3.2. Carbohydrate Synthesis and Characterization

Allyl 2,3-di-*O*-benzoyl-4,6-*O*-di-*tert*-butylsilyl-α-D-galactosyl-(1→2)-[2,3-di-*O*-benzoyl-4,6-*O*-di-*tert*-butylsilylene-α-D-galactosyl-(1→6)]-3,4-*O*-isopropylidene-β-D-galactoside (**3**): To a solution of the known galactoside acceptor **1** [53] (165 mg, 0.63 mmol) and trichloroacetimidate donor **2** [54,55] (896 mg, 1.34 mmol) in anhydrous DCM (12 mL), freshly activated crushed molecular sieves (4 Å) were added and the mixture stirred under Ar for 1 h at 0 °C. Then, TMSOTf (12 µL, 66 μmol) was added dropwise, the reaction mixture was continuously stirred for 45 min at 0 °C, and finally quenched with Et_3_N (250 µL, 1.79 mmol). The mixture was diluted with DCM, the molecular sieves were filtered off, and the mixture was washed with water and brine. The organic layer was dried over MgSO_4_, concentrated, and purified by flash column chromatography on silica gel (EtOAc/hexanes = 1:6) to afford the fully protected trisaccharide **3** (584 mg, 72%) as a white powder. *R*_f_ 0.28 (EtOAc/hexanes = 1:4). ${\left[\alpha \right]}_{D}^{26}$ + 65.9 (c = 0.08 in CH_2_Cl_2_). ^1^H NMR (400 MHz, CDCl_3,_ 300K) δ 8.04–7.93 (m, 8H, arom.), 7.60–7.46 (m, 4H, arom.), 7.45–7.31 (m, 8H, arom.), 5.79–5.70 (m, 2H), 5.67–5.56 (m, 3H), 5.49 (m, 1H, OCH_2_C*H*=CH_2_), 5.33 (d, *J* = 3.2 Hz, 1H), 4.95 (d, *J* = 17.1 Hz, 1H), 4.89–4.75 (m, 3H), 4.35–3.90 (m, 11H), 3.85–3.83 (m, 1H), 3.76–3.62 (m, 2H), 3.59–3.52 (m, 1H), 1.43 (s, 3H, CC*H*_3_), 1.12 (s, 18H, 2 × *t*Bu-Si), 1.07 (s, 3H, CC*H*_3_), 0.96 (s, 18H, 2 × *t*Bu-Si) ppm. ^13^C NMR (101 MHz, CDCl_3,_ 300K) δ 166.2 (2 × C=O), 166.0 (C=O), 165.7 (C=O), 133.3 (CH, arom., OCH_2_*C*H=CH_2_), 133.2 (CH, arom.), 133.1 (CH, arom.), 133.0 (CH, arom.), 130.0 (C, arom.), 129.8 (C, arom.), 129.8 (CH, arom.), 129.7 (CH, arom.), 129.6 (CH, arom.), 129.5 (C, arom.), 128.4 (CH, arom.), 128.3 (CH, arom.), 117.9 (C), 117.7 (CH_2_), 110.0 (C), 101.3 (CH, C-1), 96.4 (CH, C-1), 96.1 (CH, C-1), 78.0 (CH), 75.7 (CH), 73.6 (CH), 71.5 (CH), 71.3 (2 × CH), 70.8 (CH), 70.7 (CH), 70.2 (CH_2_), 68.7 (CH), 68.5 (CH), 67.0 (CH), 66.9 (2 × CH_2_), 66.7 (CH_2_), 66.6 (CH), 28.0 (CH_3_), 27.5 (CH_3_, *t*Bu), 27.3 (CH_3_, *t*Bu), 27.2 (CH_3_, *t*Bu), 26.1 (CH_3_), 23.3 (C), 23.2 (C), 20.8 (C), 20.7 (C) ppm. HR ESI-TOF-MS *m/z* calcd for C_68_H_88_NaO_20_Si_2_ [M+Na]^+^: 1303.5305, found: 1303.5257; see Figures S1–S6 in the Supplementary Materials.

Allyl 2,3-di-*O*-benzoyl-α-D-galactosyl-(1→2)-[2,3-di-*O*-benzoyl-α-D-galactosyl-(1→6)]-3,4-*O*-isopropylidene-β-D-galactoside (**4**): The fully protected trisaccharide **3** (120 mg, 0.09 mmol) was dissolved in 12 mL of anhydrous THF in a plastic conical tube and cooled to 0 °C. Then, 120 μL of HF-pyr (70%) was added and stirred for 30 min at 0 °C, and then 1 h at rt under Ar. The reaction mixture was cooled again to 0°C and quenched with saturated NaHCO_3_ solution. Finally, the mixture was extracted with EtOAc, washed with water and brine, dried over MgSO_4_, concentrated, and purified by flash column chromatography on silica gel (EtOAc/hexanes = 4:1) to furnish the desilylated trisaccharide **4** (85 mg, 90%) as a white powder. *R_f_* 0.26 (EtOAc/hexanes = 4:1). ${\left[\alpha \right]}_{D}^{26}$ + 162.7 (c = 0.14 in CH_2_Cl_2_). ^1^H NMR (400 MHz, CDCl_3,_ 300K) δ 8.03–7.93 (m, 8H, arom.), 7.55–7.43 (m, 4H, arom), 7.41–7.29 (m, 8H, arom.), 5.77–5.60 (m, 5H), 5.48 (m, 1H, OCH_2_C*H*=CH_2_), 5.36 (s, 1H), 4.96 (dd, *J* = 17.2, 1.3 Hz, 1H), 4.88 (d, *J* = 10.4 Hz, 1H), 4.45 (d, *J* = 11.6 Hz, 2H), 4.31 (t, *J* = 3.6 Hz, 1H), 4.17 (d, *J* = 8.4 Hz, 1H), 4.15–4.04 (m, 3H), 4.01–3.89 (m, 6H), 3.89–3.83 (m, 1H), 3.74 (dd, *J* = 9.9, 5.8 Hz, 1H), 3.64–3.50 (m, 2H), 3.40–3.26 (m, 2H, -OH), 2.86–2.76 (m, 1H, -OH), 1.45 (s, 3H, CC*H*_3_), 1.06 (s, 3H, CC*H*_3_), 0.91–0.79 (m, 1H, -OH) ppm. ^13^C NMR (101 MHz, CDCl_3,_ 300K) δ 166.1 (C=O), 166.0, (2 × C=O), 165.8 (C=O), 133.5 (CH, arom.), 133.4 (CH, arom.), 133.3 (CH, arom., OCH_2_*C*H=CH_2_), 129.9 (CH, arom.), 129.8 (CH, arom.), 129.7 (C, arom.), 129.6 (C, arom.), 129.5 (C, arom.), 129.4 (C, arom.), 128.6 (CH, arom.), 128.5 (CH, arom.), 117.8 (CH_2_), 110.3 (C), 101.3 (CH, C-1), 96.8 (CH, C-1), 96.7 (CH, C-1), 78.1 (CH), 77.4 (CH), 73.5 (CH), 71.4 (CH), 71.0 (CH), 70.9 (CH), 70.4 (CH), 70.2 (CH_2_), 69.8 (CH), 69.3 (CH), 69.0 (2 × CH), 68.6 (CH), 66.6 (CH_2_), 63.8 (CH_2_), 63.2 (CH_2_), 28.1 (CH_3_), 26.1 (CH_3_) ppm. HR ESI-TOF-MS *m/z* calcd for C_52_H_56_NaO_20_ [M+Na]^+^: 1023.3263, found: 1023.3252, see Figures S7–S12 in the Supplementary Materials.

Allyl 2,3-di-*O*-benzoyl-α-D-galactosyl-(1→2)-[2,3-di-*O*-benzoyl-α-D-galactosyl-(1→6)]-β-D-galactoside (**5**): To a solution of trisaccharide **4** (260 mg, 0.26 mmol) in DCM (8 mL), H_2_O (1.0 mL) and TFA (1.0 mL) were consecutively added, and the mixture was vigorously stirred at rt for 30 min. After disappearance of starting material based on TLC, the resulting solution was twice co-evaporated with EtOH (10 mL). The residue was then further dried under vacuum and purified by flash column chromatography on silica gel (DCM/MeOH = 9:1) to afford the partially deprotected **5** (240 mg, 96%) as a colorless syrup. R*_f_* 0.33 (DCM/MeOH = 9:1). ${\left[\alpha \right]}_{D}^{26}$ + 215.6 (c = 0.05 in CH_2_Cl_2_). ^1^H NMR (400 MHz, methanol-d_4,_ 300K) δ 8.02-7.86 (m, 8H, arom.), 7.55–7.46 (m, 4H, arom), 7.45–7.29 (m, 8H, arom.), 5.72–5.61 (m, 5H), 5.55 (m, 1H, OCH_2_C*H*=CH_2_), 5.31 (d, *J* = 3.2 Hz, 1H), 4.94 (dd, *J* = 17.4, 1.5 Hz, 1H), 4.86–4.79 (m, 1H), 4.61 (t, *J* = 6.0 Hz, 1H), 4.37–4.27 (m, 3H), 4.10 (t, *J* = 6.0 Hz, 1H), 4.02–3.91 (m, 2H), 3.85–3.69 (m, 9H), 3.52 (dd, *J* = 12.5, 5.9 Hz, 1H) ppm. ^13^C NMR (101 MHz, methanol-d_4,_ 300K) δ 166.1 (C=O), 166.0 (2 × C=O), 165.8 (C=O), 133.7 (CH, arom.), 133.2 (CH, arom.), 133.1 (CH, arom.), 133.0 (CH, arom., OCH_2_*C*H=CH_2_), 129.7 (C, arom.), 129.6 (C, arom.), 129.5 (C, arom.), 129.4 (CH, arom.), 129.3 (CH, arom.), 129.2 (CH, arom.), 128.2 (CH, arom.), 128.1 (CH, arom.), 116.1 (CH_2_), 102.6 (CH, C-1), 96.2 (CH, C-1), 96.1 (CH, C-1), 75.6 (CH), 72.8 (CH), 72.2 (CH), 71.3 (2 × CH), 71.0 (CH), 70.32 (CH), 69.7 (CH_2_), 69.5 (CH), 69.2 (CH), 69.0 (CH), 67.7 (CH), 67.5 (CH), 66.4 (CH_2_), 61.1 (CH_2_), 61.0 (CH_2_) ppm. HR ESI-TOF-MS *m/z* calcd for C_49_H_52_NaO_20_ [M + Na]^+^: 983.2950, found: 983.2975; see Figures S13–S18 in the Supplementary Materials.

3-(Acetylthio)propyl 2,3-di-*O*-benzoyl-α-D-galactosyl-(1→2)-[2,3-di-*O*-benzoyl-α-D-galactosyl-(1→6)]-β-D-galactoside (**6**): To a solution of allyl trisaccharide **5** (230 mg, 0.24 mmol) and AIBN (20 mg, 0.12 mmol) in anhydrous THF (3 mL) under Ar, thioacetic acid (43 µL, 0.60 mmol) was added, and the mixture was stirred under water cooling (~ 25 °C) for 6 h in a Rayonet UV reactor equipped with 350 nm lamps. The solution was then co-evaporated with toluene and concentrated to near dryness. The crude product was purified by flash column chromatography on silica gel (CHCl_3_/MeOH = 14:1) to afford the acyl-protected trisaccharide **6** (188 mg, 76%) as a white solid. R*_f_* 0.33 (CHCl_3_/MeOH = 7:1). ${\left[\alpha \right]}_{D}^{26}$ + 100.1 (c = 0.07 in MeOH). ^1^H NMR (400 MHz, methanol-d_4,_ 300K) δ 8.02–7.85 (m, 8H, arom.), 7.61–7.28 (m, 12H, arom), 5.72–5.59 (m, 5H), 5.49 (s, 1H), 5.31 (d, *J* = 2.4 Hz, 1H), 4.66–4.57 (m, 1H), 4.37–4.28 (m, 2H), 4.23 (d, *J* = 7.0 Hz, 1H), 4.16–3.89 (m, 3H), 3.89-3.63 (m, 9H), 3.59–3.48 (m, 1H), 2.95–2.83 (m, 1H), 2.71–2.55 (m, 2H), 2.23 (s, 3H) ppm. ^13^C NMR (101 MHz, methanol-d_4,_ 300K) δ 195.9 (C=O), 166.1 (C=O), 166.0 (2 × C=O), 165.8 (C=O), 133.2 (CH, arom.), 133.1 (CH, arom.), 133.0 (CH, arom.), 129.7 (C, arom.), 129.6 (C, arom.), 129.5 (C, arom.), 129.3 (CH, arom.), 129.2 (CH, arom.), 128.3 (CH, arom.), 128.2 (CH, arom.), 128.1 (CH, arom.), 103.5 (CH, C-1), 96.1 (CH, C-1), 96.0 (CH, C-1), 75.6 (CH), 72.8 (CH), 72.1 (CH), 71.3 (2 × CH), 71.0 (CH), 70.2 (CH), 69.6 (CH), 69.3 (CH), 69.1 (CH), 67.7 (CH), 67.7 (CH_2_), 67.6 (CH), 66.3 (CH_2_), 61.0 (2 × CH_2_), 29.3 (CH_2_), 25.3 (CH_2_) ppm. HR ESI-TOF-MS *m/z* calcd for C_51_H_56_NaO_21_S [M+Na]^+^: 1059.2932, found: 1059.2925; see Figures S19–S22 in the Supplementary Materials.

3-Thiopropyl α-D-galactosyl-(1→2)-[α-D-galactosyl-(1→6)]-β-D-galactoside [**(G11_S_)_2_**]: The acyl-protected trisaccharide **6** (188 mg, 0.18 mmol) was dissolved in 20 mL of anhydrous 0.25 M NaOMe, and stirred for 2 h under Ar at rt. The solution was then neutralized with Amberlyst-15, filtered through celite, concentrated, and finally dissolved in water and lyophilized. Initially, the unprotected mercaptopropyl trisaccharide **G11_SH_** was produced, which oxidizes by handling on air within hours to the disulfide **(G11_S_)_2_** (118 mg, quant.) as an off-white solid. *R*_f_ 0.25 [(*i*PrOH/H_2_O = 5:1 w/3 drops AcOH (27 μL added to 12 mL of eluent)]. ${\left[\alpha \right]}_{D}^{26}$ + 61.6 (c = 0.03 in H_2_O). ^1^H NMR (400 MHz, D_2_O, 300 K) δ 5.34 (d, 2H, *J* = 3.8 Hz, H-1), 4.93 (d, 2H, *J* = 2.7 Hz, H-1), 4.50 (d, 2H, *J* = 7.9 Hz, H-1), 4.14–4.29 (m, 2H), 3.53–4.06 (m, 42H), 3.31 (s, 4H), 2.71–2.88 (m, 4H), 1.95–2.11 (m, 4H), 1.87 (s, 12H) ppm. ^13^C NMR (101 MHz, D_2_O, 300K) δ 103.3 (C-1), 98.1 (C-1), 97.9 (C-1), 73.0 (CH), 71.5 (CH), 70.9 (CH), 70.6 (CH), 69.5 (CH), 69.3 (CH), 69.2 (2 × CH), 69.1 (CH), 68.7 (CH_2_), 68.3 (CH), 68.2 (CH), 66.3 (CH_2_), 61.2 (CH_2_), 60.9 (CH_2_), 34.3 (CH_2_), 28.4 (CH_2_), 23.3 (CH) ppm. HR ESI-TOF-MS *m/z* calcd for C_42_H_74_NaO_32_S_2_ [M+Na]^+^: 1177.3502, found: 1177.3310; see Figures S23–S28 in the Supplementary Materials.

### 3.3. Conjugation of the Glycan with Maleimide-Derivatized Bovine Serum Albumin (BSA)

The kit for the conjugation of the thiol-containing glycan **G11_SH_** to BSA derivative **7** (Imject Maleimide-Activated BSA, catalogue number 77116, Thermo Fisher Scientific) and the conjugation procedure was performed according to the manufacturer’s instructions and as previously published [39,46]. Briefly, tris(2-carboxyethyl)phosphine hydrochloride (TCEP·HCl, 0.8 mg, 2.8 μmol) was dissolved in 250 μL conjugation buffer (83 mM sodium phosphate buffer, 0.1 M EDTA, 0.9 M sodium chloride, and 0.02% sodium azide, pH 7.2). The TCEP·HCl solution was added to a 1.5 mL microcentrifuge tube that contained sugar-disulfide **(G11_S_)_2_** (2.7 mg, 2.4 μmol), and the mixture was agitated in a shaker for 30 min to furnish sugar-thiol **G11_SH_**. An aliquot of 10 μL (0.11 mg of **G11_SH_**) was set aside for the colorimetric determination of the thiol concentration. The maleimide-activated BSA (**7**) (2 mg, 15–25 moles of maleimide/mole BSA) was reconstituted with 200 μL of Ultrapure MilliQ water to produce a 10 mg/mL solution. The trisaccharide solution was added to the reconstituted maleimide-activated BSA (**7**) and incubated at rt for 2–3 h in a shaker. Then, 18.3 μL (corresponding to the same quantity of **G11_SH_**, if no conjugation had occurred) was removed from the conjugation mixture to determine the concentration of unreacted thiol. This aliquot was diluted to 2.75 mL with reaction buffer (0.1 M sodium phosphate, pH 8.0, containing 1 mM EDTA), combined with 50 μL of Ellman’s reagent [5,5′dithiobis-(2-nitrobenzoic acid), DTNB] solution (4 mg DTNB in 1 mL reaction buffer), and reacted for 15 min at rt. The absorbance at 412 nm was measured in a UV-vis spectrophotometer. The thiol concentration was determined using the molar extinction coefficient of 2-nitro-5-thiobenzoic acid (TNB), ε = 14,150 M^−1^ cm^−1^, and the amount of sugar conjugated (typically, 2.0 μmol) was calculated.

The conjugation mixture was diluted with ultrapure water to a volume of 1 mL and desalted using an Amicon Ultra 3K centrifugal filter and was centrifuged for 20 min at 4000× *g*, rt. The mixture was washed with 1 mL of ultrapure water three times following the same procedure. The tube with the filtrate was then removed, and 500 µL of ultrapure water was added to the NGP11b solution remaining in the filter. Since a small amount of aggregation can occur, the solution/suspension was transferred onto a 2-mL Zeba spin desalting column (7K MWCO, Thermo Fisher Scientific), provided in the kit, that had been previously washed with 1 mL of ultrapure pure water 4 times by centrifugation at 1000× *g* for 2 min, rt. This procedure removed all salts and aggregated protein. The filtrate was lyophilized, and the solid NGP can be stored at −50 °C for at least 6 months. In our hands, this combination of filtration and size-exclusion chromatography avoids or minimizes aggregation of the NGP. To determine the NGP11b quantity, a solution of 1–2 mg of it in 1–3 mL of ultrapure water was prepared, and the concentration was determined with a Pierce BCA Protein Assay Reagent kit using a spectrophotometer at 562 nm.

### 3.4. MALDI-TOF-MS

To determine the mass of the maleimide-derivatized BSA (**7**) and NGP11b, 1 µL of **7** (125 µg/mL) was combined in a 1.5 mL microcentrifuge tube with 1 µL NGP11b (670 µg/mL) and 2 µL of matrix (20 mg/mL sinapinic acid, 50% acetonitrile, 0.1% TFA). Two microliters of the sample-matrix mixture were spotted onto a 48-well steel MALDI plate and allowed to crystallize at room temperature for approximately 20 min. The mass spectra were acquired using a Shimadzu MALDI-8020 mass spectrometer set to linear mode with dithering at a scan range of 10,000 to 100,000 *m/z*. Data acquisition included a laser power of 110, laser repetition rate (Hz) of 50, accumulated shots 5, blast shots 2, profiles at 200, pulse extraction set to 66431, and a blanking mass of 15,000. Spectra were processed by Threshold Apex set at constant threshold, Gaussian smoothing, smoothing filter width of 200, and peak width of 2. MS-grade BSA standard (Thermo Scientific Pierce Bovine Serum Albumin Standard Ampules, 2 mg/mL) was used for calibration and internal references set at [BSA + H]^+^ = 66,431 and [BSA + 2H]^2+^ = 33,216, with a 5 ppm mass tolerance.

### 3.5. Purification of tGPI-MUC from T. cruzi Colombiana (DTU TcI) and (DTU TcII) Y Strains

tGPI-MUC preparations were obtained from *T. cruzi* mammal-dwelling infective trypomastigote stage of Colombiana (DTU *T. cruzi* I, or TcI) and Y (DTU TcII, or TcII) strains [44], as previously described [39].

### 3.6. Human Serum and Plasma Samples

Individual serum samples (*n* = 58) from adult patients diagnosed with CCD were obtained from the Centro Regional de Investigación en Salud Pública, Instituto Nacional de Salud Pública, Tapachula, Mexico (*n* = 42) and the Universidad Central de Venezuela (ICV), Facultad de Medicina, Instituto de Medicina Tropical, Caracas, Venezuela (*n* = 16). Negative control serum samples of healthy individuals from a nonendemic area (*n* = 15) were acquired from the Gulf Coast Regional Blood Center (Houston, TX, USA), and kindly donated by Dr. Dapeng Zhou (formerly at MD Anderson Cancer Center, USA, currently at Tongji University, Shanghai, China). In addition, umbilical cord plasma samples (*n* = 27), with both negative real-time PCR and non-conventional serology for CCD (with nine in-house *T. cruzi* antigens; to be published elsewhere) were obtained from the Sierra Medical Center, Sierra East Medical Center, and Providence Memorial Hospital, El Paso, Texas. Serum samples (*n* = 6) were also obtained from CCD patients from the Hospital Clinic, Barcelona, Spain prior to or after the standard-of-care chemotherapy for CCD (BZN, 150 mg, twice a day). All human samples were obtained under approved IRB protocols, as described in detail in the Institutional Review Board Statement and Informed Consent Statement sections below.

### 3.7. CL-ELISA

CL-ELISA was performed as described in [39] to assess the specificity of IgG from patients infected with CD against NGP11b in comparison to purified TcI tGPI-MUC (from *T. cruzi* Colombiana strain) and TcII tGPI-MUC (from *T. cruzi* Y strain). These antigens were immobilized in 96-well Nunc MaxiSorp polystyrene microplates for 16 h at 4 °C in 200 mM carbonate-bicarbonate buffer, pH 9.6 (CBB). After antigen loading, the plates were blocked with 200 μL per well of phosphate-buffered saline (PBS) containing 1% BSA (PBS-B) and incubated for 1 h at 37 °C. Pools of CCD sera (CCDSP) (*n* = 10), normal human sera (NHSP) (*n* = 10), individual CCD patient sera (*n* = 58), or negative non-CCD control (NC) plasma (*n* = 27) from healthy individuals were analyzed in triplicate, at 1:400 or 1:800 dilution, in PBS-B plus 0.05% Tween 20 (PBS-TB). Then, plates were sequentially incubated with 50 μL goat anti-human IgG (H + L) secondary antibody, biotinylated (catalog # 31770, 1.5 mg/mL, Thermo Fisher Scientific), diluted at 1:10,000 in PBS-TB, and 50 μL Pierce High Sensitivity Neutravidin-Horseradish Peroxidase (catalog # 31030, 1.0 mg NeutrAvidin/mL, Thermo Fisher Scientific), and diluted at 1:5,000 in PBS-TB. All incubation steps were performed for 1 h at 37 °C, followed by washing three times with 200 μL PBS, containing 0.05% Tween 20 (PBS-T), using an automatic microplate washer dispenser (EL406 BioTek, Agilent). Finally, plates were developed with 50 μL SuperSignal ELISA Pico Chemiluminescent Substrate (containing the Pico Stable Peroxide Solution and SuperSignal ELISA Pico Luminol Enhancer) (catalogue 37069 or 37070, Pierce, Thermo Fisher Scientific) diluted in CBB/0.1% BSA in a 1:1:8 ratio (*v/v/v*), and the relative luminescence units (RLU) were measured using a Luminoskan Accent Luminometer (Thermo Fisher Scientific).

The levels of IgG antibodies to NGP11b (50 ng/well), TcI tGPI-MUC (30 ng/well), and TcII tGPI-MUC (30 ng/well) in individual sera of CCD patients (*n* = 58) or negative non-CCD control plasma (*n* = 27) from healthy individuals were evaluated. Each serum (at 1:800 dilution) or umbilical cord plasma (at 1:400 dilution, considering a 1:2 dilution of the plasma) sample was diluted in PBS-TB and tested in technical triplicates. The incubation steps were performed exactly as described above for the cross-titration. To normalize the RLU readings and minimize the interplate variations, the results were expressed as CL-ELISA titers. These were calculated by dividing the mean RLU value of each test sample (TS) (${\overset{–}{x}}_{\mathrm{TS}}$) in triplicate by the cutoff value, which was calculated as follows: cutoff = ${\overset{–}{x}}_{\mathrm{NC}}$ + SD*f*, where ${\overset{–}{x}}_{\mathrm{NC}}$ is the mean RLU value of nine technical replicates of the NC (plasma pool from healthy individuals) per microplate; and SD*f* is the standard deviation multiplier calculated based on the number of negative control replicates per microplate, as described in [56]. Here, using a confidence level [1-α] of 99.5% and 9 controls per microplate, the SD*f* was 4.335.

### 3.8. Statistical Analysis

Cross-titration curves (antigen concentration vs. serum dilution) were compared using ordinary one-way ANOVA with the Holm–Sidak comparison test, with a single pooled variance. For the comparison of the serum reactivity of distinct serum panels with different antigens, the Kruskal–Wallis test followed by Dunn’s multiple-comparison posttest was used. Statistical significance was set at the conventional 5% level (*p* < 0.05) and all analyses were performed using GraphPad Prism version 9.0 (GraphPad Software, San Diego, CA). Finally, multiple logistic regression models followed by ROC curve analyses were performed on normalized CL-ELISA titer values to establish the sensitivity, specificity, and other performance parameters obtained, using GraphPad Prism v. 9.0.

## 4. Conclusions

A branched trisaccharide with two terminal, nonreducing α-Gal units (Galα(1,2)[Galα(1,6)]Galβ-(CH_2_)_3_SH) was synthesized and conjugated with BSA to produce the neoglycoprotein NGP11b, which was serologically evaluated as a BMK for CD diagnosis and for early assessment of drug efficacy in patients. The strong and dose-dependent IgG Ab responses of sera from CCD patients and little to no reactivity of sera from healthy individuals suggested that Galα(1,2)[Galα(1,6)]Galβ glycotope may be an immunodominant structure in infective forms of *T. cruzi*, or a mimic thereof. These results, together with the previous observation that CCD patient sera and NHS showed only a small difference in Ab reactivity to an NGP with α-Gal monosaccharide moieties [46], support that α-Gal is part of larger glycotope structures, such as Galα(1,2)[Galα(1,6)]Galβ.

Using a large cohort of 85 individual sera from CCD patients and healthy donors, we demonstrated by CL-ELISA with NGP11b that we can place *T. cruzi*-positive and *-*negative sera into two distinct groups. These results showed that NGP11b was of superior diagnostic value when compared to the purified TcI or TcII tGPI-MUC and, therefore, has the potential to replace tGPI-MUC as a BMK for CCD in serological assays. By means of the reversed glycomics approach, we also demonstrated for the first time that a synthetic glycotope likely present on *T. cruzi* tGPI-MUC can potentially be used for the early assessment of parasiticidal drug efficacy in CCD patients. We demonstrated that the seroreactivity to the double α-Gal glycotope of NGP11b was significantly reduced 24 months following BZN chemotherapy in all six CCD patients. Nevertheless, further validation through a prospective, well-designed phase II clinical trial will determine the true value of NGP11b as a prognostic tool.

In summary, our results corroborate the antigenicity and specificity of the α-Gal epitopes in NGP11b. Due to significant differential antibody reactivities between sera of CCD patients and healthy individuals, NGP11b seems suitable as a diagnostic CCD BMK, and also shows potential for monitoring the follow-up serological status of CCD patients after chemotherapy. Current efforts are focused on the discovery and immunological evaluation of glycan-based BMKs that produce even greater antibody-differential reactivities between *T. cruzi*-positive and -negative sera and on extending chemotherapy studies to longer periods of follow-up with various NGPs for a more accurate assessment of treatment outcomes.

## Figures and Tables

**Table 1:** Immunoreactivity of sera of CCD patients and plasma of negative controls with TcI tGPI-MUC, TcII tGPI-MUC, and NGP11b.

Disease/Control	*n*	NGP11b		TcI tGPI-MUC		TcII tGPI-MUC	
		Positive	Negative	Positive	Negative	Positive	Negative
Chronic Chagas disease	58	55 (95.8%)	3 (5.2%)	51 (87.9%)	7 (12.1%)	32 (55.2%)	26 (44.8%)
Healthy control ^a^	27	0	27 (100%)	0	27 (100%)	0	27 (100%)

^a^ Plasma from umbilical cord samples with negative real-time PCR and non-conventional serology (with nine in-house *T. cruzi* antigens) for CCD.

**Table 2:** Sensitivity, specificity, and other diagnostic parameters of NGP11b, TcI tGPI-MUC, and TcII tGPI-MUC, in the comparison of CCD vs. healthy individuals.

Parameter ^a,b^	NGP11b	TcI tGPI-MUC	TcII tGPI-MUC
	*%*		
Sensitivity	94.8	87.9	55.2
Specificity	100.0	100.0	100.0
FPR	0.0	0.0	0.0
PPV	100.0	100.0	100.0
NPV	90.0	79.4	50.9
Accuracy	96.6	92.4	76.6

^a^ Calculated based on CCD (*n* = 58) and NC (*n* = 27) immunoreactivity with NGP11b, TcI tGPI-MUC, and TcII tGPI-MUC (Figure 2B). ^b^ Sensitivity = [true positive (TP)/TP + false negative (FN)] × 100. Specificity = [true negative (TN)/TN + false positive (FP)] × 100. False-positive rate (FPR) = 100 − specificity. Positive predictive value (PPV) = TP/TP + FP. Negative predictive value (NPV) = TN/TN + FN; Accuracy = (TN + TP)/(TN + TP + FN + FP).

## Data Availability

Some of the results described here have been reported in Dr. Alba L. Montoya’s dissertation, 2020, University of Texas at El Paso. All data produced in the present study are available upon reasonable request to the authors.

## Supplementary Materials

The following are available online at https://www.mdpi.com/article/10.3390/molecules27175714/s1: Figures S1–S6: ^1^H, ^13^C, COSY, and HSQC NMR spectra as well as HR ESI-TOF and simulated MS of compound **3**; Figures S7–S12: ^1^H, ^13^C, COSY, and HSQC NMR spectra, as well as HR ESI-TOF and simulated MS of compound **4**; Figures S13–S18: ^1^H, ^13^C, COSY, and HSQC NMR spectra, as well as HR ESI-TOF and simulated MS of compound **5**; Figures S19–S22: ^1^H and ^13^C NMR spectra as well as HR ESI-TOF and simulated MS of compound **6**; Figures S23–28: ^1^H, ^13^C, COSY, and HSQC NMR spectra, as well as HR ESI-TOF and simulated MS of compound **(G11_S_)_2_**_._

## Sample Availability

Owing to the minimal amount of NGP11b synthesized and the unique nature of all deidentified sera or plasma used here, these samples will be available only for collaborative studies until further notice by the corresponding senior authors of this study.

## Abbreviations

**Ab**, antibody; **AcSH**, thioacetic acid; **AIBN**, α,α‘-azoisobutyronitrile; **AUC**, area under the curve; **BCA**, bicinchoninic acid; **BSA**, bovine serum albumin; **BNZ**, benznidazole; **BMK**, biomarker; **CBB**, carbonate-bicarbonate buffer; **CCD**, chronic Chagas disease; **CCDSP**, chronic Chagas disease serum pool; **CD**, Chagas disease; **Ch anti-α-Gal Abs**, anti-α-Gal antibodies from Chagas disease patients; **CI**, confidence interval; **CL-ELISA**, chemiluminescent enzyme-linked immunosorbent assay; **CS**, conventional serology; **DCM**, dichloromethane; **DTNB**, 5,5′dithiobis-(2-nitrobenzoic acid); **DTU**, discrete typing unit; **EDTA**, ethylenediaminetetraacetic acid; **EtOAc**, ethyl acetate; **FPR**, false-positive rate; **Gal**, galactopyranose; **Gal*****f***, β-galactofuranose; **α1,3-GalT**, α1,3-galactosyltransferase; **GIPL**, glycoinositolphospholipid; **GlcNAc**, *N*-acetylglucosamine; **GNP**, gold glyconanoparticle; **GPI**, glycosylphosphatidylinositol; **GPI-MUC**, glycosylphosphatidylinositol-anchored mucin(s); **GU**, glycan unit(s); **HR ESI-TOF-MS**, high-resolution electrospray ionization time-of-flight mass spectrometry; **IgG**, immunoglobulin G; **MALDI-TOF-MS**, matrix-assisted laser desorption ionization time-of-flight mass spectrometry; **Mal-BSA**, maleimide-bovine serum albumin; **MS**, mass spectrometry; **MWCO**, molecular weight cutoff; **NC**, negative control; **NFX**, nifurtimox; **NGP**, neoglycoprotein; **NHS**, normal human serum; **NHSP**, normal human serum pool; **NMR**, nuclear magnetic resonance; **NPV**, negative predictive value; **NTD**, neglected tropical disease; **PBS**, phosphate-buffered saline; **PCR**; polymerase chain reaction; **PPV**, positive predictive value; **pyr**, pyridine; **RLU**, relative luminescence units; **ROC**, receiver-operating characteristics; **rt**, room temperature; **TBN**, 2-nitro-5-thiobenzoic acid; **TcI**–**TcVI**, *T. cruzi* genotypes I–VI; **Tcbat**, *T. cruzi* genotype bat; **TCEP-HCl**, tris(2-carboxyethyl)phosphine hydrochloride; **TcMUC**, GPI-anchored mucin(s); ***T. cruzi***, *Trypanosoma cruzi*; **TFA**, trifluoroacetic acid; **tGPI-MUC**, glycosylphosphatidylinositol-anchored mucins from the trypomastigote form of *T. cruzi*; **TLC**, thin-layer chromatography; **THF**, tetrahydrofuran; **TMSOTf**, trimethylsilyl trifluoromethanesulfonate; **TS**, test sample.
