# Supplementary material for: A Branched and Double Alpha-Gal-Bearing Synthetic Neoglycoprotein as a Biomarker for Chagas Disease
Source: Molecules. 2022 Sep 5;27(17):5714. doi: 10.3390/molecules27175714 (PMC9457857; doi:10.3390/molecules27175714)
Supplement: Supplementary file 1 [file molecules-27-05714-s001.zip › molecules-1862968-supplementary.pdf]

SUPPLEMENTARY MATERIALS

A Branched and Double Alpha-Gal-Bearing Synthetic Neoglycoprotein as a Biomarker for Chagas Disease

Alba L. Montoya 1,†, Elisa G. Carvajal 1,†, Uriel Ortega-Rodriguez 2,‡, Igor L. Estevao 2,‡, Roger A. Ashmus 1, Sohan R. Jankuru 1, Susana Portillo 2, Cameron C. Ellis 2, Colin D. Knight 2, Julio Alonso-Padilla 3,4, Luis Izquierdo 3,4, Maria-Jesus Pinazo 3,4,5, Joaquim Gascon 3,4,5, Veronica Suarez 2, Douglas M. Watts 2, Iliana R. Malo 6, Janine M. Ramsey 6, Belkisyolé Alarcón De Noya 7, Oscar Noya 8, Igor C. Almeida 2,* and Katja Michael 1,*

1 Department of Chemistry and Biochemistry, Border Biochemical Research Center, The University of Texas at El Paso, El Paso, TX 79968, USA

2 Department of Biological Sciences, Border Biochemical Research Center, The University of Texas at El Paso, El Paso, TX 79968, USA

3 Barcelona Institute for Global Health (ISGlobal), Barcelona 08003, Spain

4 Consorcio Centro de Investigación Biomédica en Red (CIBER) de Enfermedades Infecciosas, Instituto de Salud Carlos III (CIBERINFEC, ISCIII), Madrid 28029, Spain

5 Hospital Clínic de Barcelona, Barcelona 08036, Spain

6 Centro Regional de Investigación en Salud Pública, Instituto Nacional de Salud Pública, Tapachula 30700, Chiapas, Mexico

7 Sección de Inmunología, Instituto de Medicina Tropical, Universidad Central de Venezuela, Caracas 1041, Venezuela

8 Seccion de Biohelmintiasis, Instituto de Medicina Tropical, Universidad Central de Venezuela, Caracas 1041, Venezuela

* Correspondence: icalmeida@utep.edu (I.C.A.); kmichael@utep.edu (K.M.)

† These authors contributed equally to this work.

‡ These authors contributed equally to this work.

TABLE OF CONTENTS

General Information	S3
Abbreviations used	S3
Figure S1: ^{1}H NMR spectrum of compound **3**	S4
Figure S2: ^{13}C NMR spectrum of compound **3**	S5
Figure S3: COSY NMR spectrum of compound **3**	S6
Figure S4: HSQC NMR spectrum of compound **3**	S7
Figure S5: HR ESI-TOF mass spectrum of compound **3**	S8
Figure S6: Actual and simulated HR ESI-TOF mass spectrum of compound **3**	S9
Figure S7: ^{1}H NMR spectrum of compound **4**	S10
Figure S8: ^{13}C NMR spectrum of compound **4**	S11
Figure S9: COSY NMR spectrum of compound **4**	S12
Figure S10: HSQC NMR spectrum of compound **4**	S13
Figure S11: HR ESI-TOF mass spectrum of compound **4**	S14
Figure S12: Actual and simulated HR ESI-TOF mass spectrum of compound **4**	S15
Figure S13: ^{1}H NMR spectrum of compound **5**	S16
Figure S14: ^{13}C NMR spectrum of compound **5**	S17
Figure S15: COSY NMR spectrum of compound **5**	S18
Figure S16: HSQC NMR spectrum of compound **5**	S19
Figure S17: HR ESI-TOF mass spectrum of compound **5**	S20
Figure S18: Actual and simulated HR ESI-TOF mass spectrum of compound **5**	S21
Figure S19: ^{1}H NMR spectrum of compound **6**	S22
Figure S20: ^{13}C NMR spectrum of compound **6**	S23
Figure S21: HR ESI-TOF mass spectrum of compound **6**	S24
Figure S22: Actual and simulated HR ESI-TOF mass spectrum of compound **6**	S25
Figure S23: ^{1}H NMR spectrum of compound **$(G11_S)_2$**	S26
Figure S24:^{13}C NMR spectrum of compound **$(G11_S)_2$**	S27
Figure S25: COSY NMR spectrum of compound **$(G11_S)_2$**	S28
Figure S26: HSQC NMR spectrum of compound **$(G11_S)_2$**	S29
Figure S27: HR ESI-TOF mass spectrum of compound **$(G11_S)_2$**	S30
Figure S28: Actual and simulated HR ESI-TOF mass spectrum of compound **$(G11_S)_2$**	S31

GENERAL INFORMATION

^{1}H and ^{13}C Nuclear Magnetic Resonance (NMR) spectra were recorded on a Bruker Advance III HD 400 MHz NMR spectrometer at 400 and 101 MHz, respectively. Tetramethylsilane (δ 0.00 ppm) was used as a reference to determine the chemical shift δ [ppm] as an internal standard in $CDCl_3$ and CD_3OD or relative to the $CDCl_3$ signal (δ 77.0 ppm) in ^{13}C NMR spectra. When spectra were measured in D_2O, a solution of tetramethylsilane in CDCl3 in a sealed capillary was used as an external standard for calibration. The coupling constants J [Hz] were measured from one-dimensional ^{1}H-NMR spectra. Full or partial assignments were made by standard COSY, HSQC, and TOCSY experiments. High-resolution mass spectra were recorded using electrospray ionization mass spectrometry (ESI-MS) on a high-resolution JEOL AccuTOF mass spectrometer.

ABBREVIATIONS USED

COSY	Correlated Spectroscopy
HR ESI-TOF-MS	High Resolution Electrospray Ionization Time-of-Flight Mass Spectrum
HSQC	Heteronuclear Single Quantum Coherence
m/z	Mass-to-Charge Ratio
NMR	Nuclear Magnetic Resonance

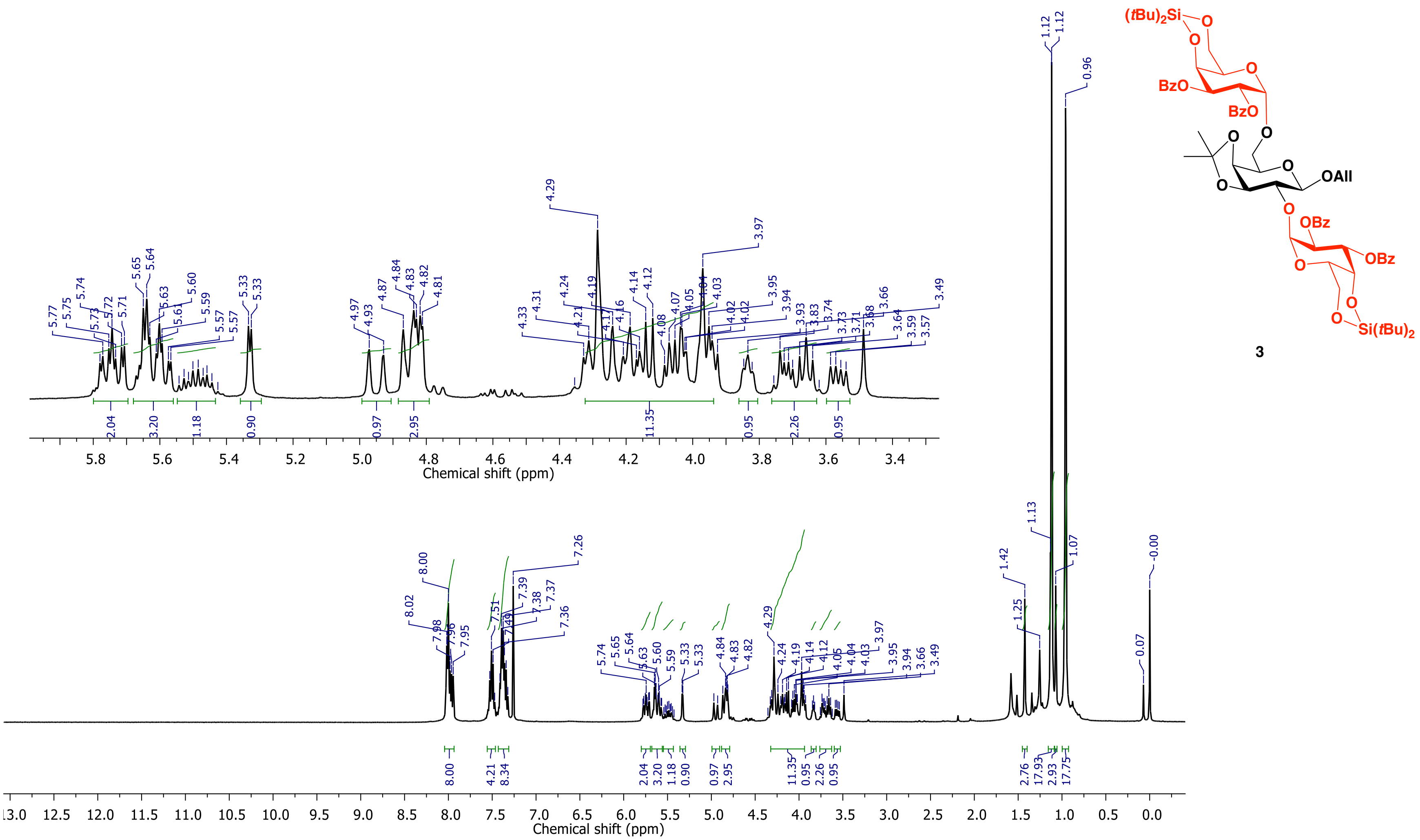

Figure S1: ^{1}H NMR spectrum of compound **3**, (400 MHz, in $CDCl_3$)

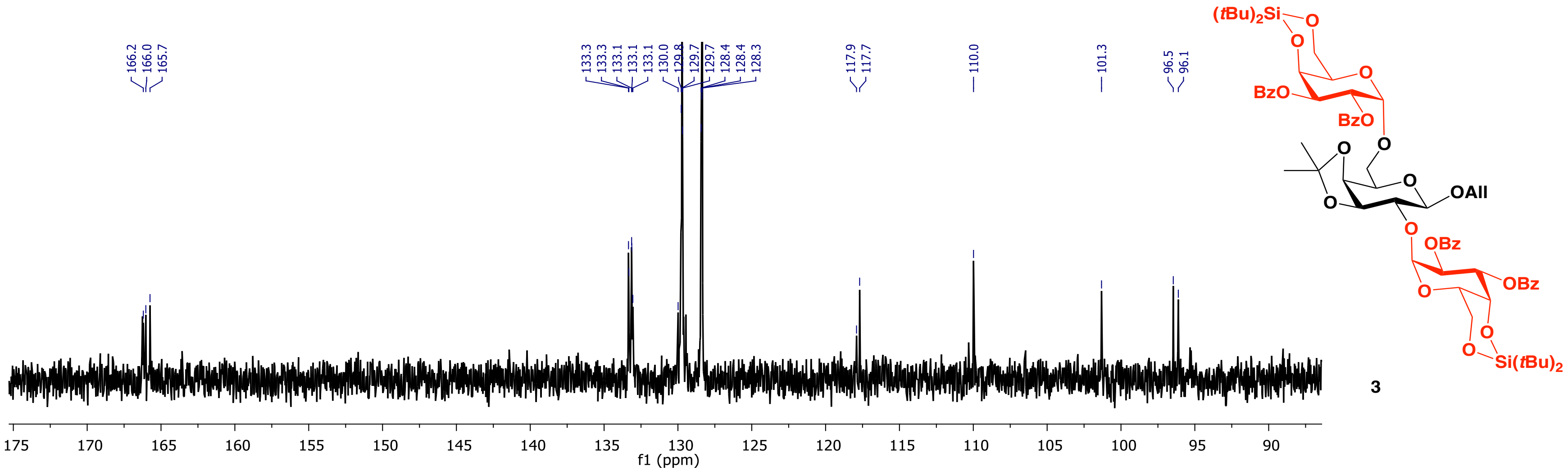

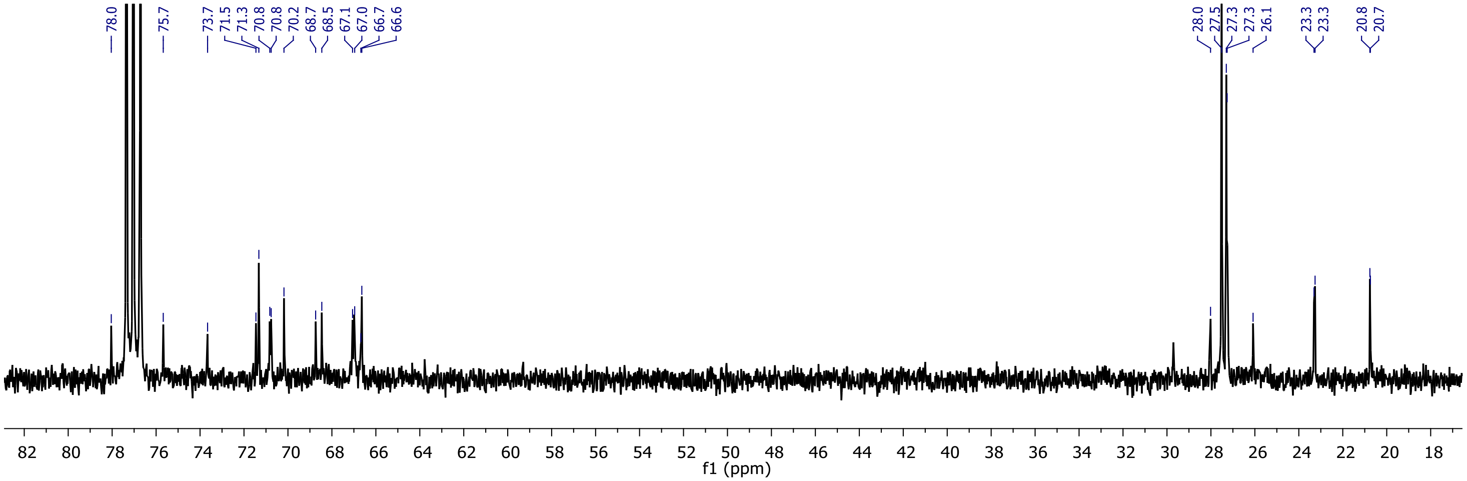

Figure S2: ^{13}C NMR spectrum of compound **3** (101 MHz, in $CDCl_3$)

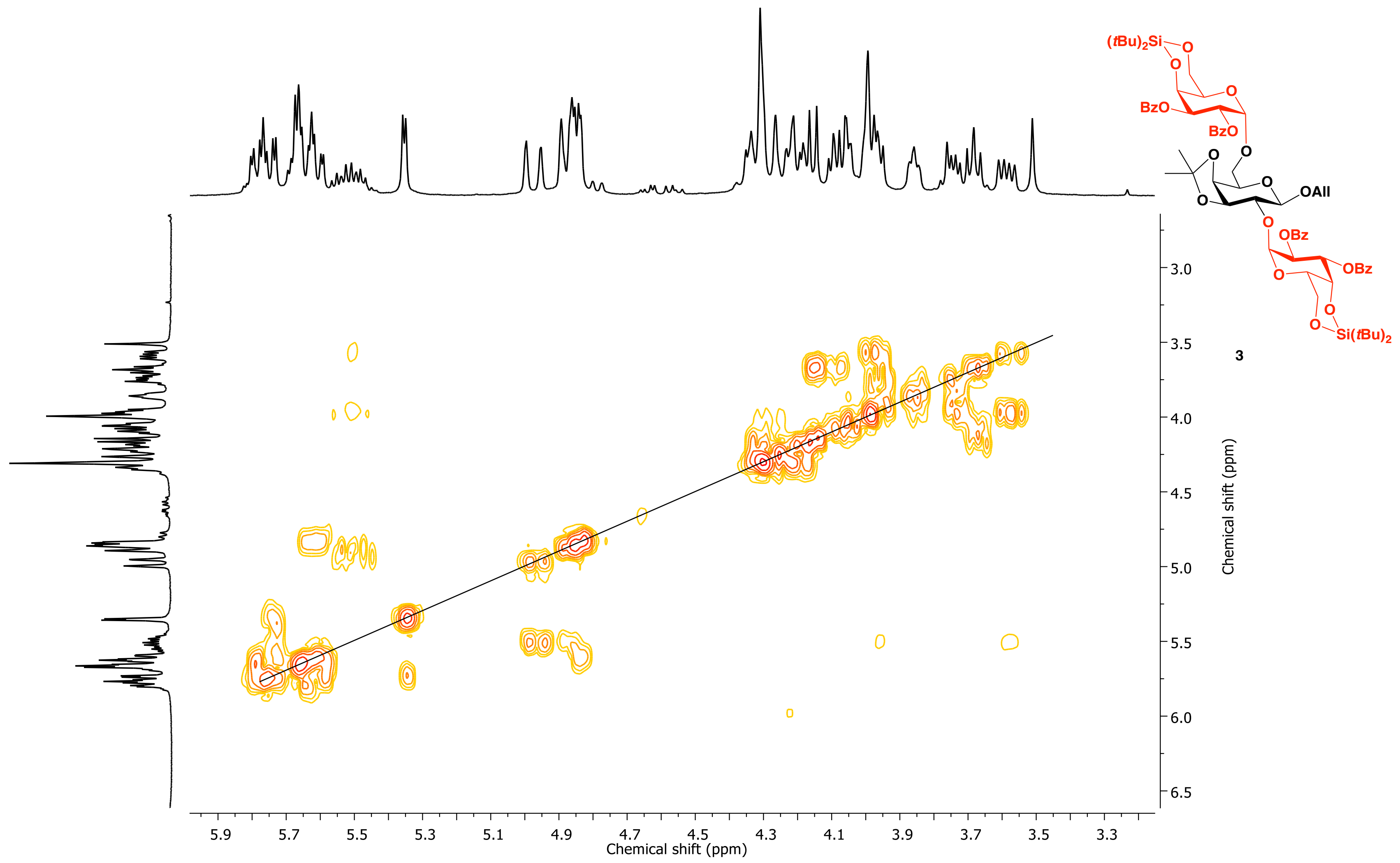

Figure S3: COSY NMR spectrum of compound **3** (400 MHz, in $CDCl_3$)

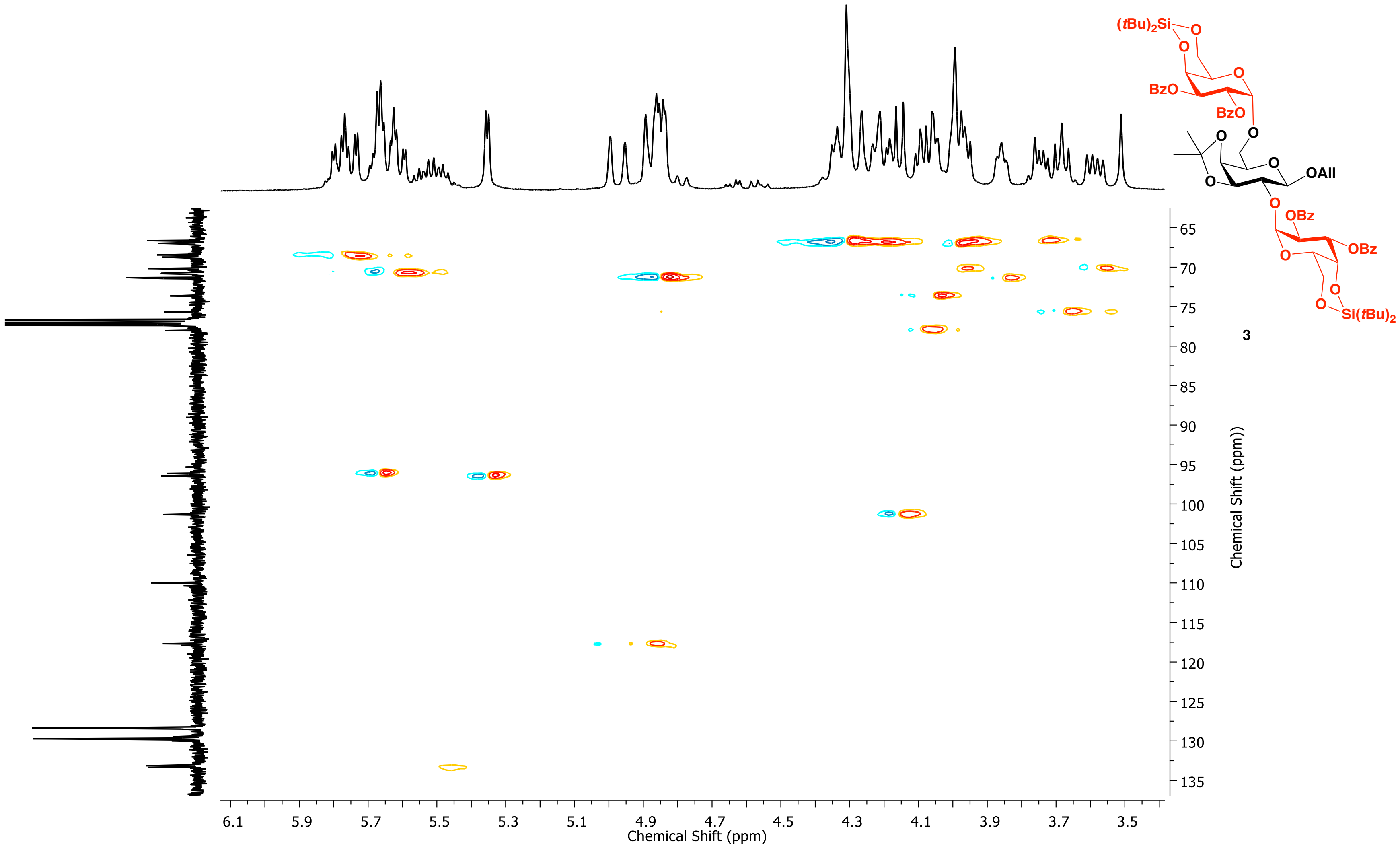

Figure S4: HSQC NMR spectrum of compound **3** (in $CDCl_3$)

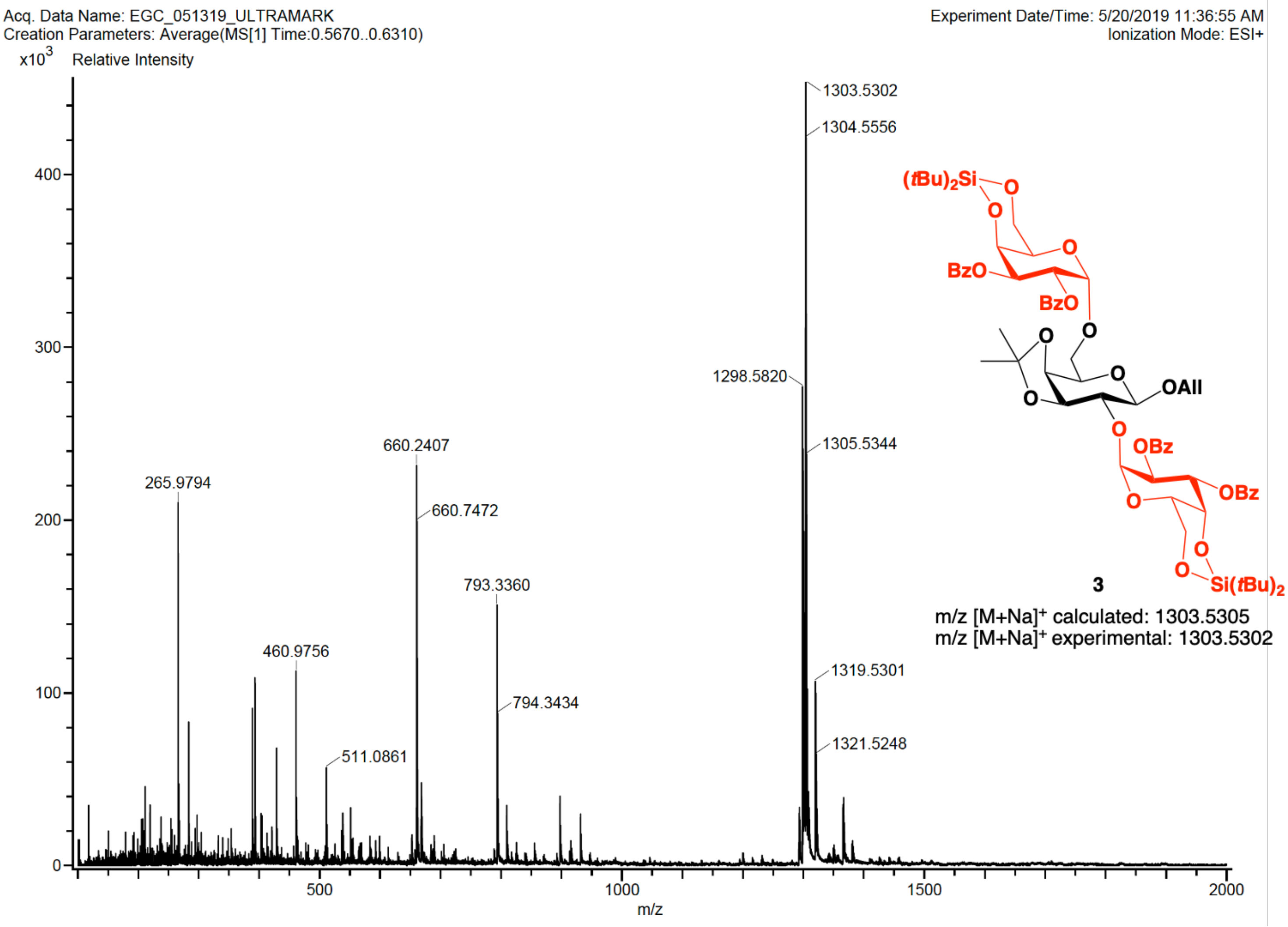

Figure S5: HR ESI-TOF mass spectrum of compound **3**

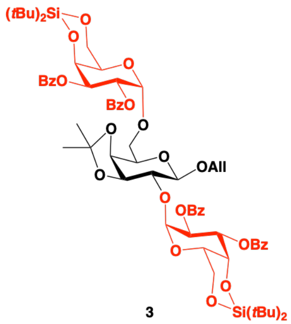

3

m/z $[M+Na]^+$ calculated: 1303.5305
m/z $[M+Na]^+$ experimental: 1303.5302

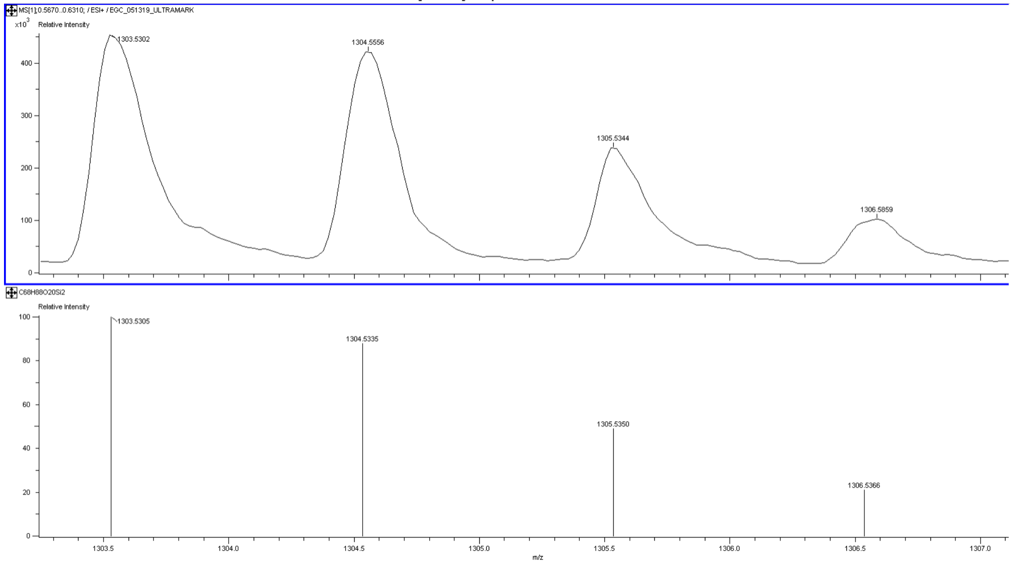

Figure S6: Actual and simulated HR ESI-TOF mass spectrum of compound **3**

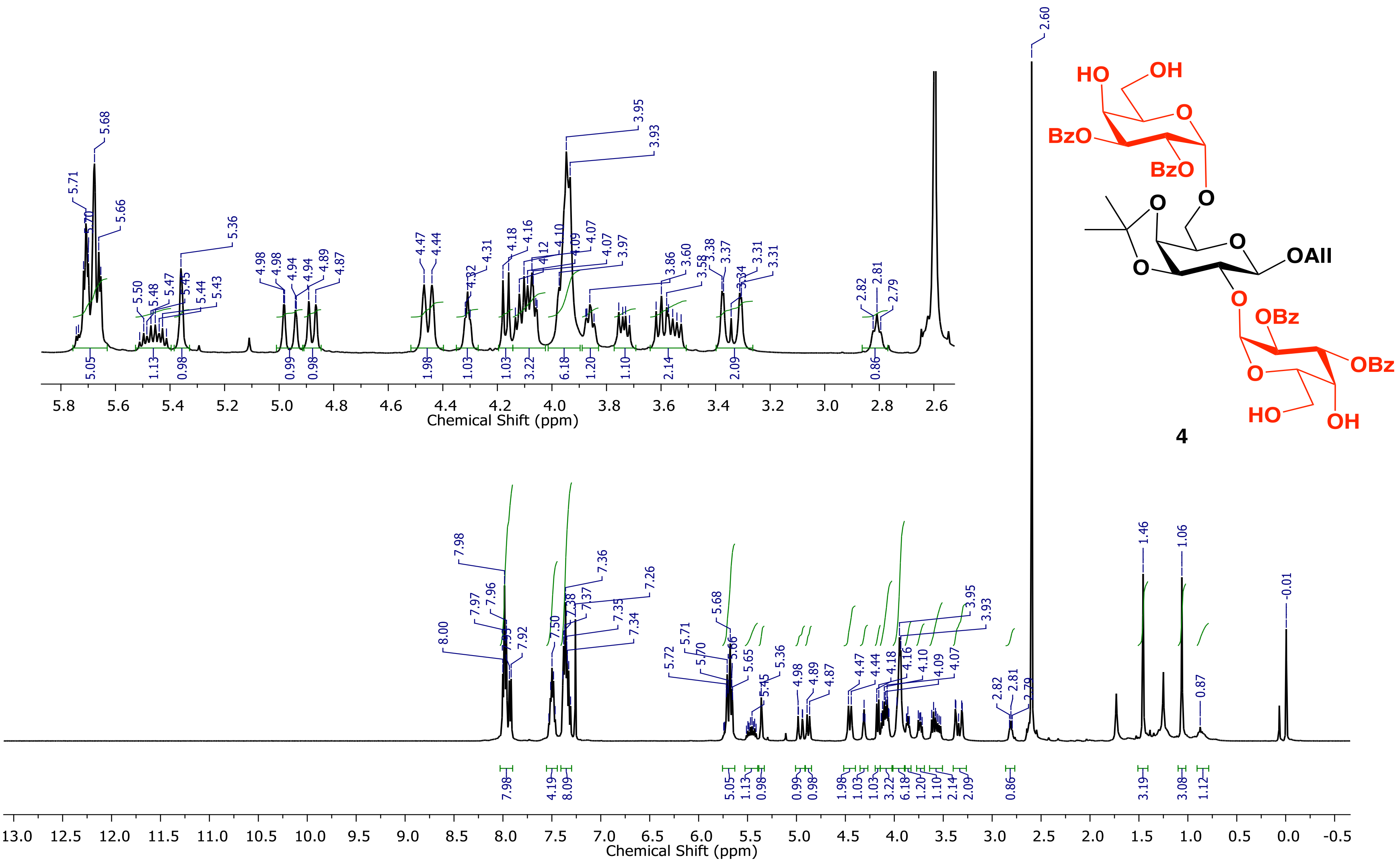

4

Figure S7: ^{1}H NMR spectrum of compound **4** (400 MHz, in $CDCl_3$)

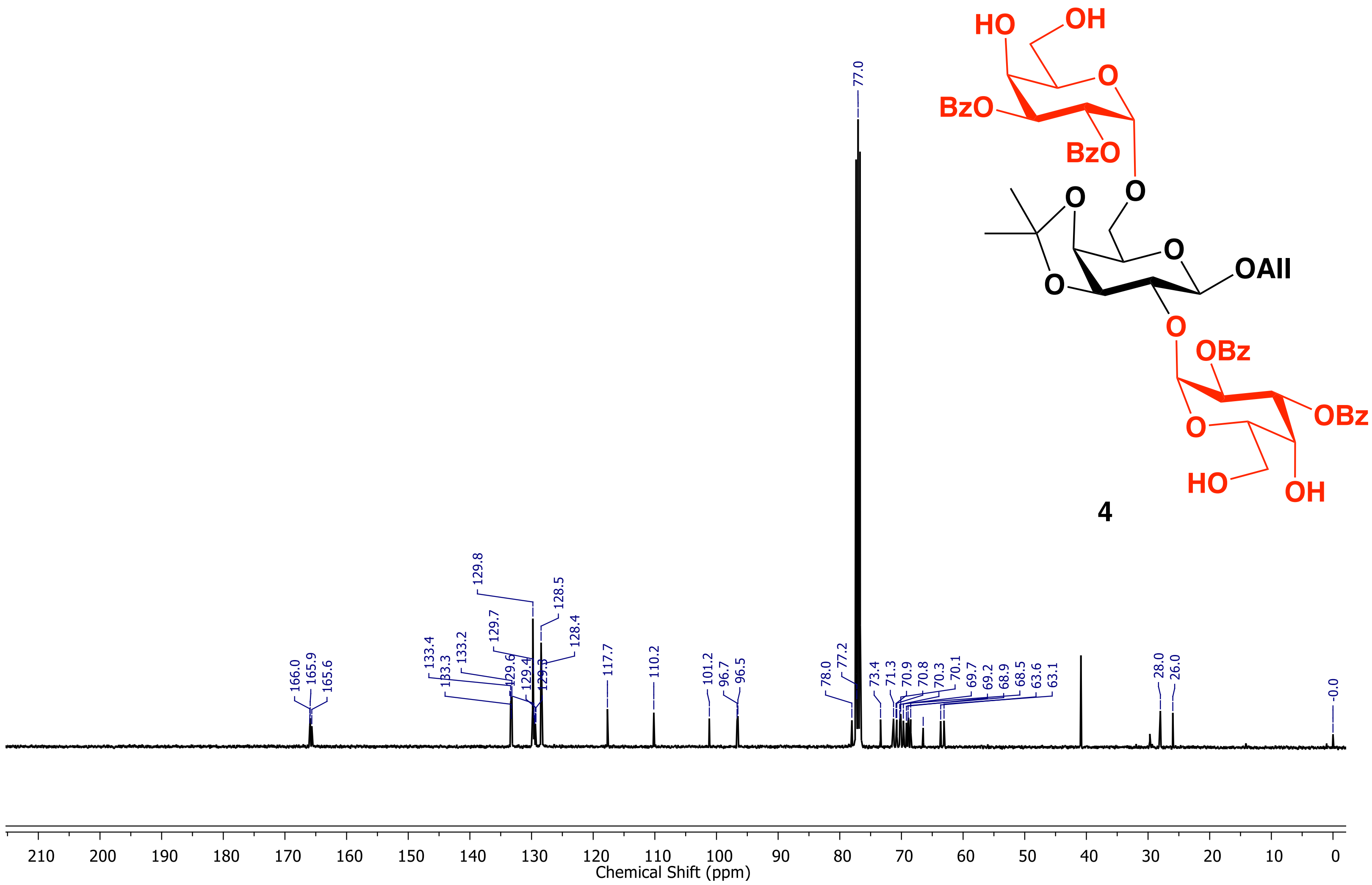

Figure S8: ^{13}C NMR spectrum of compound **4** (101 MHz, in $CDCl_3$)

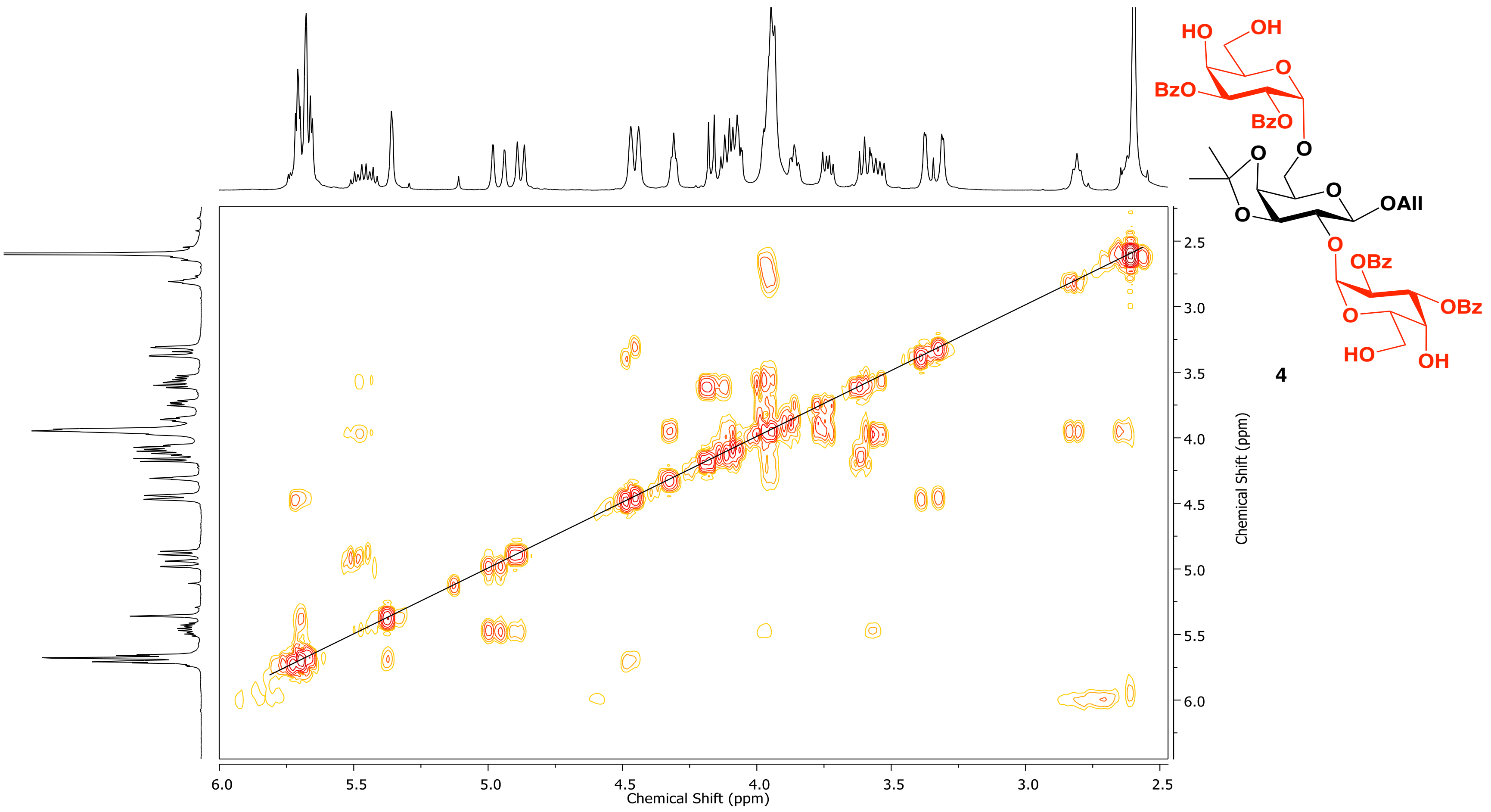

Figure S9: COSY NMR spectrum of compound **4** (in $CDCl_3$)

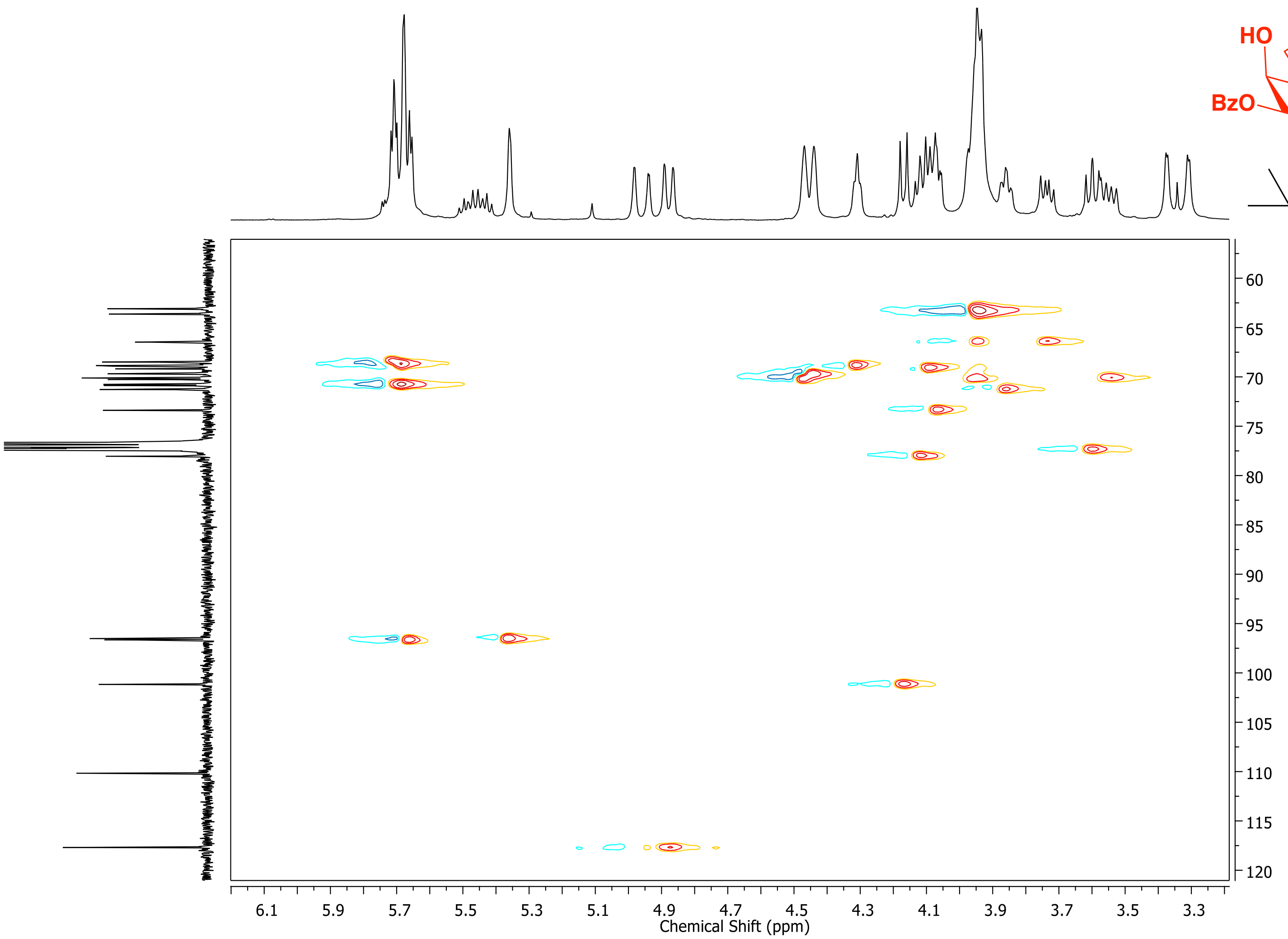

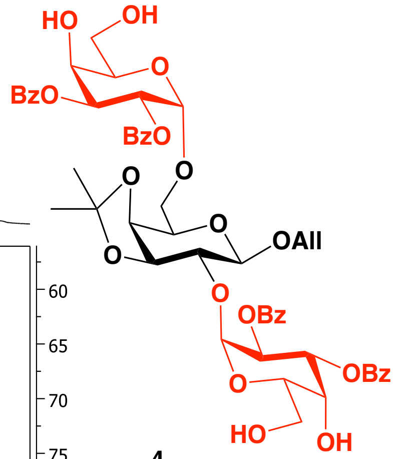

4

Figure S10: HSQC NMR spectrum of compound **4** (in $CDCl_3$)

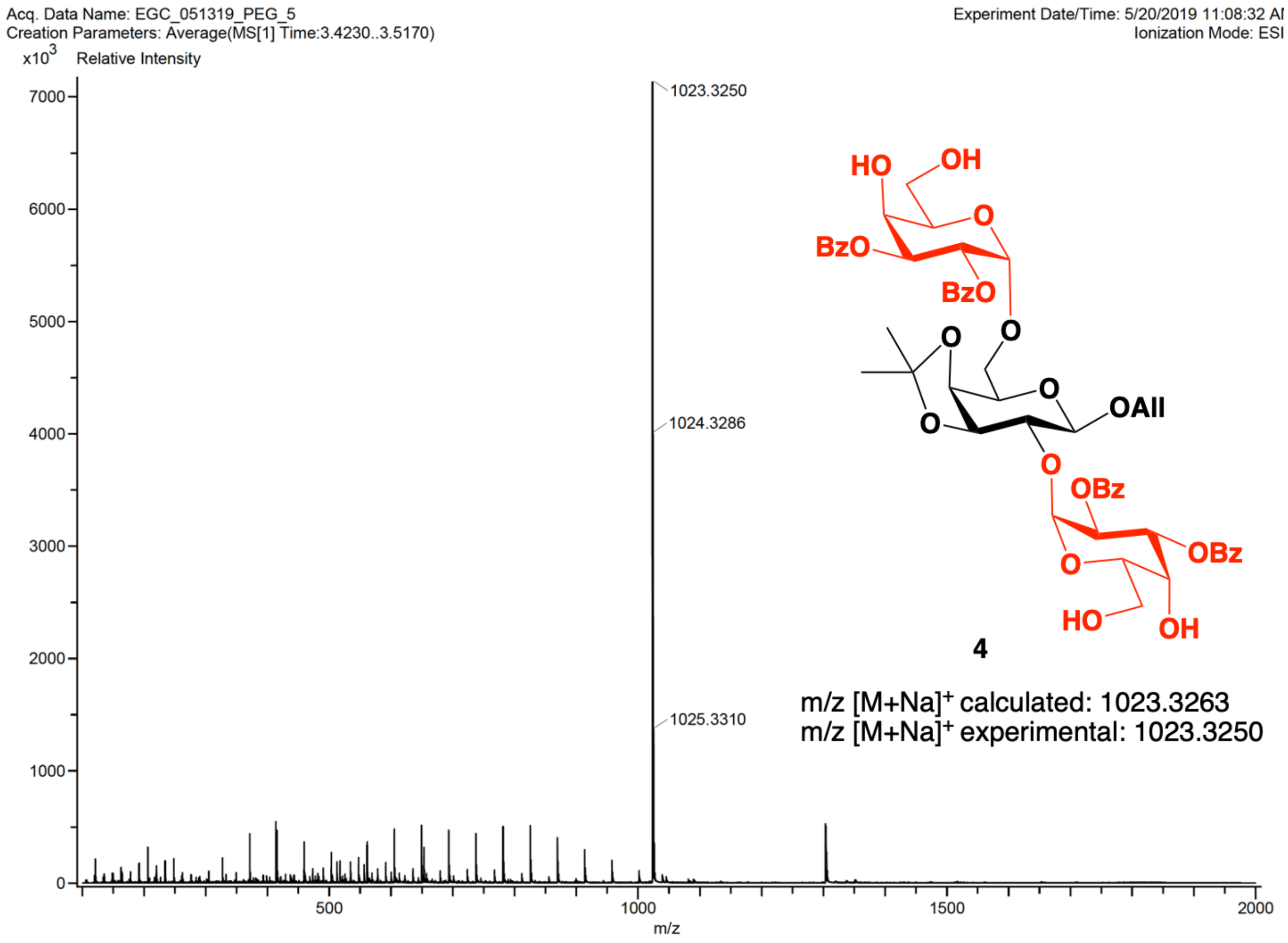

Figure S11: HR ESI-TOF mass spectrum of compound **4**

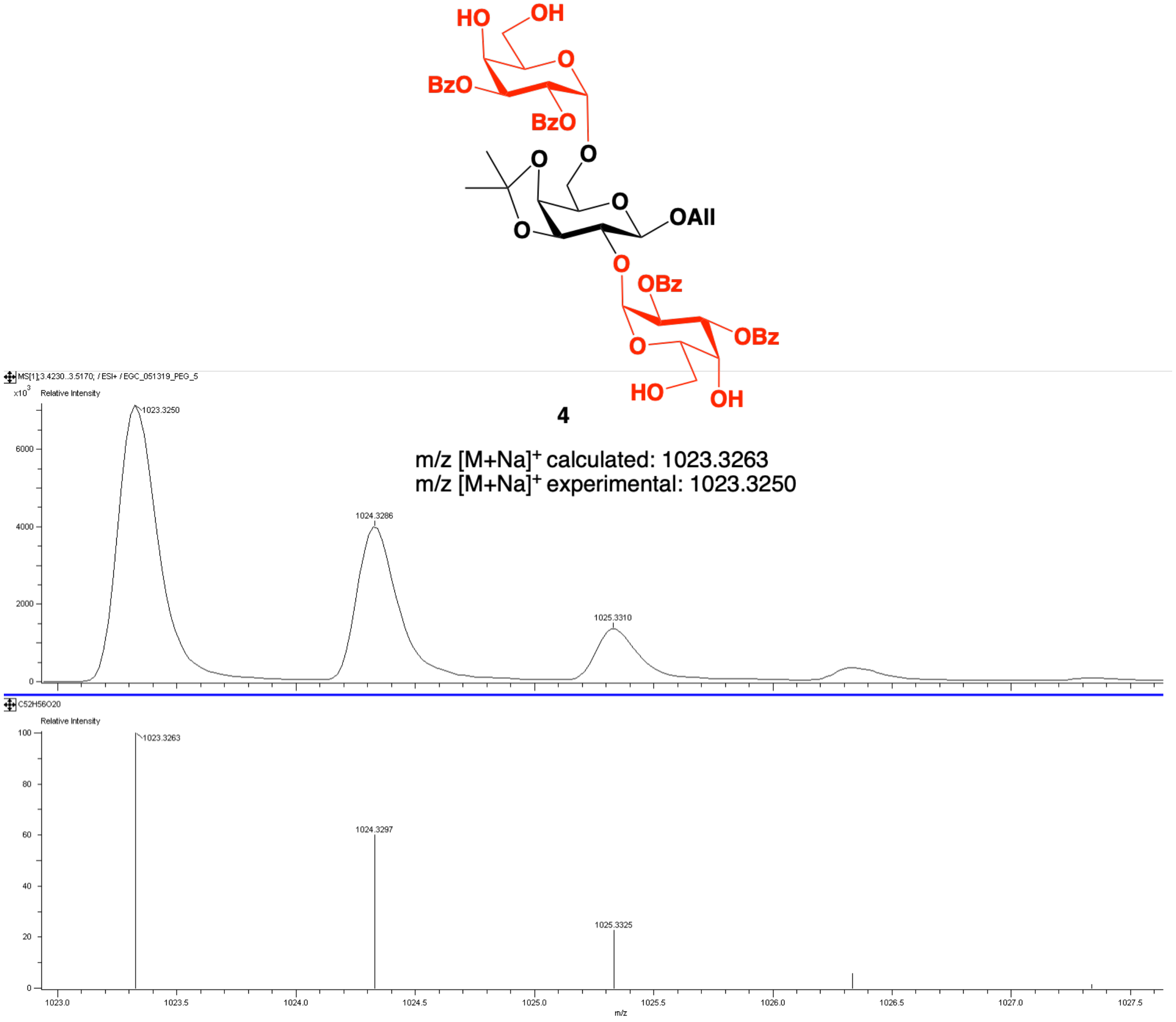

Figure S12: Actual (top) and simulated (bottom) HR ESI-TOF mass spectrum of compound **4**

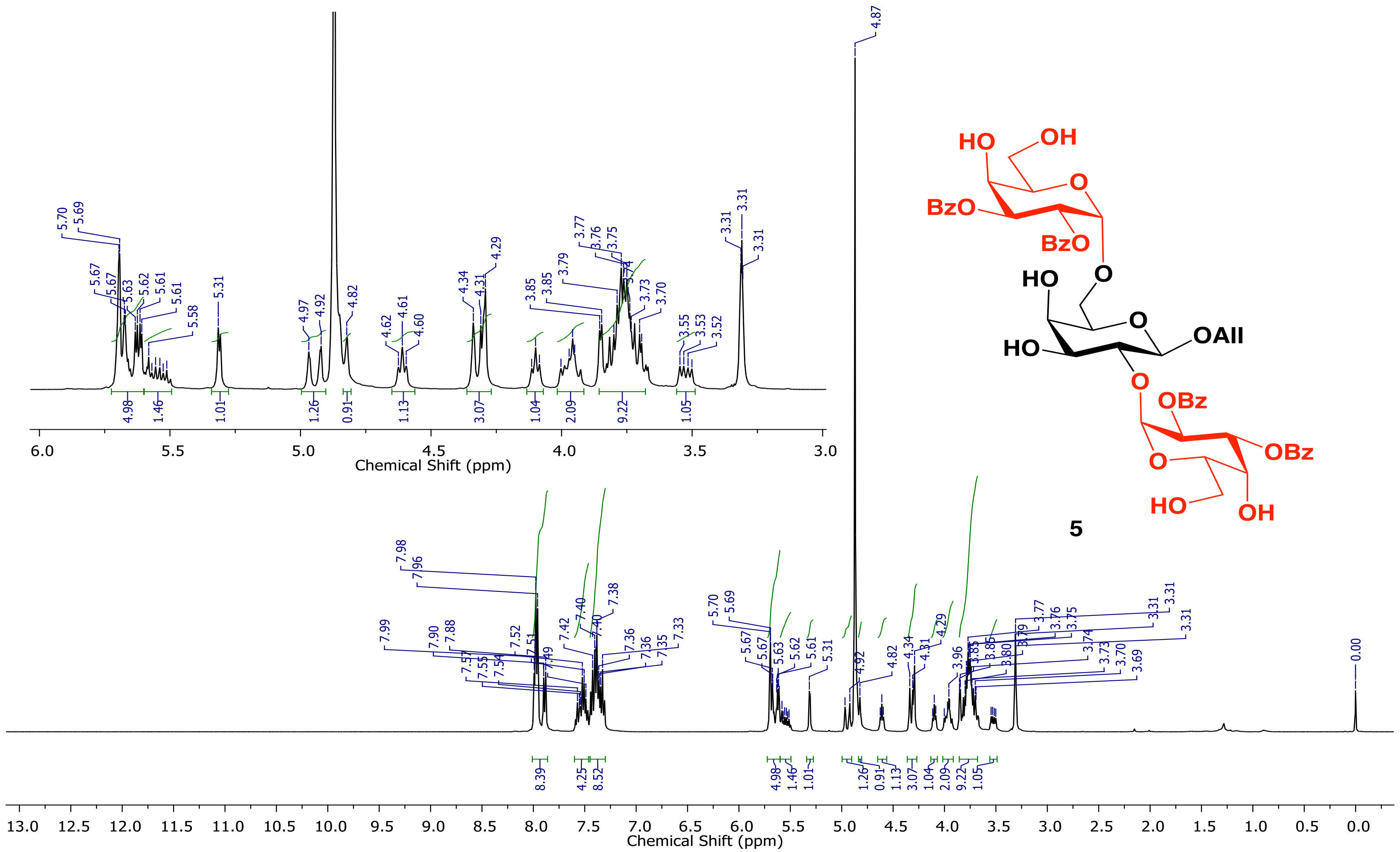

Figure S13: ^{1}H NMR spectrum of compound **5** (400 MHz, in methanol-d_4)

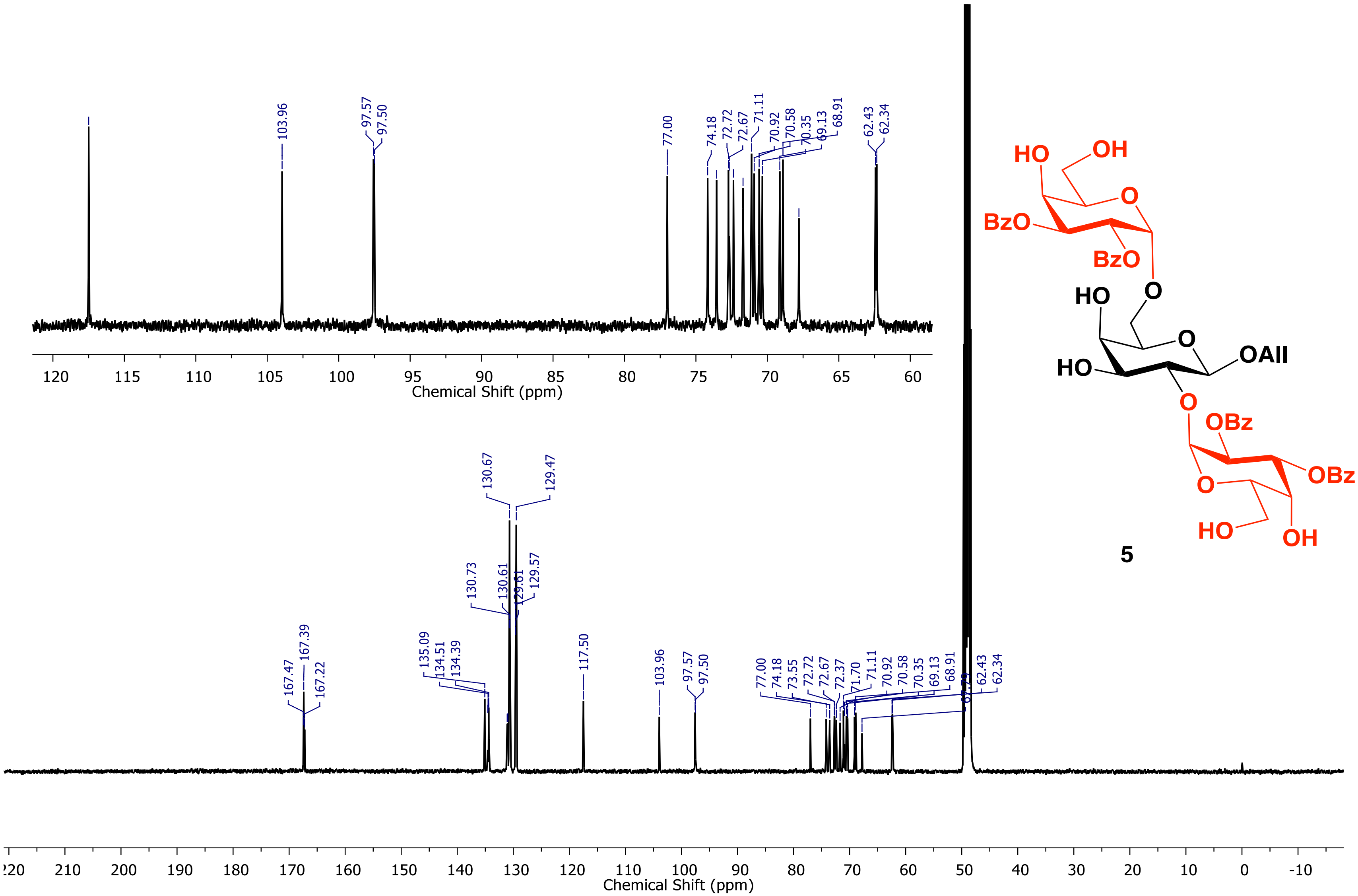

Figure S14: ^{13}C NMR spectrum of compound **5** (101 MHz, in methanol-d_4)

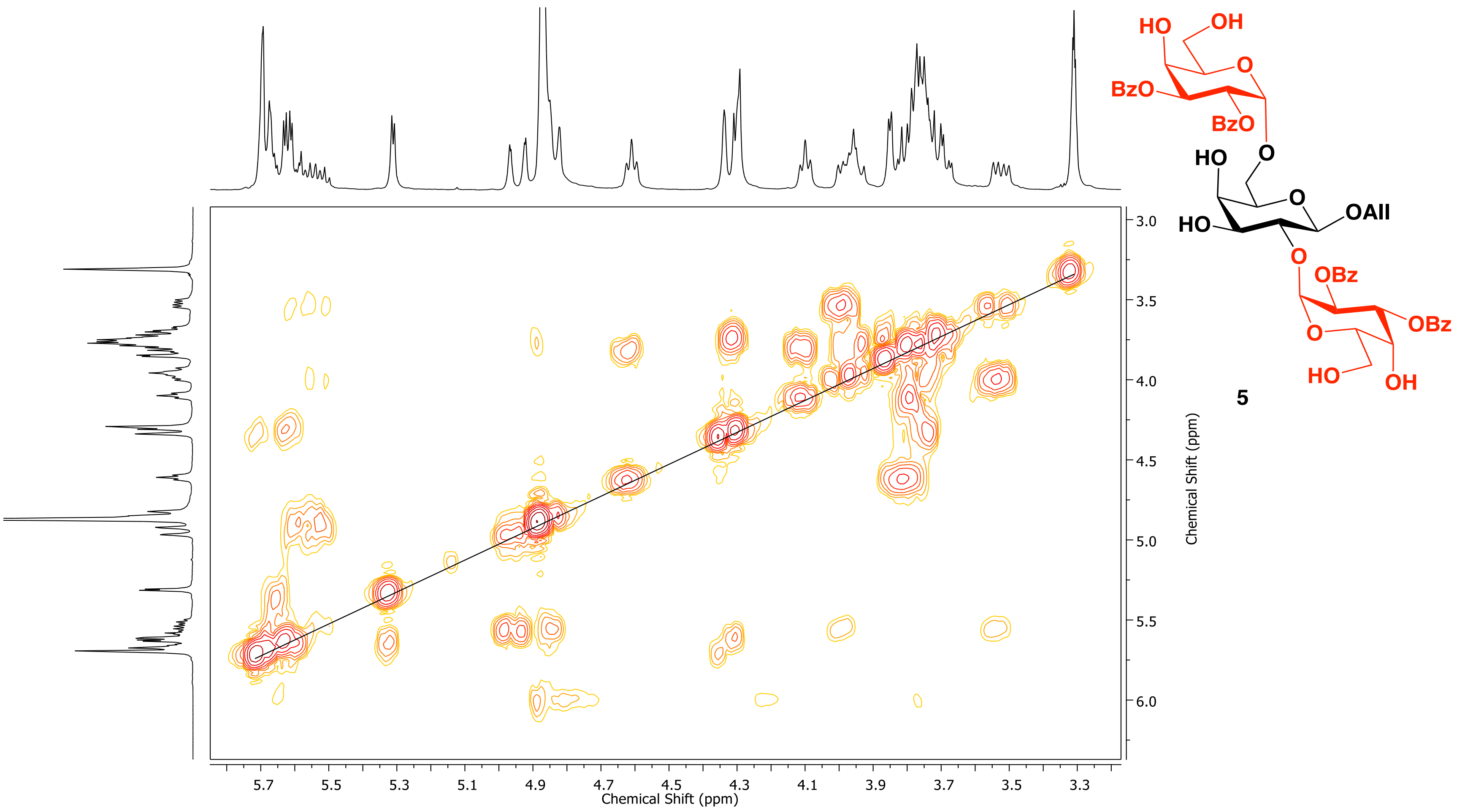

Figure S15: COSY NMR spectrum of compound **5** (in methanol-d_4)

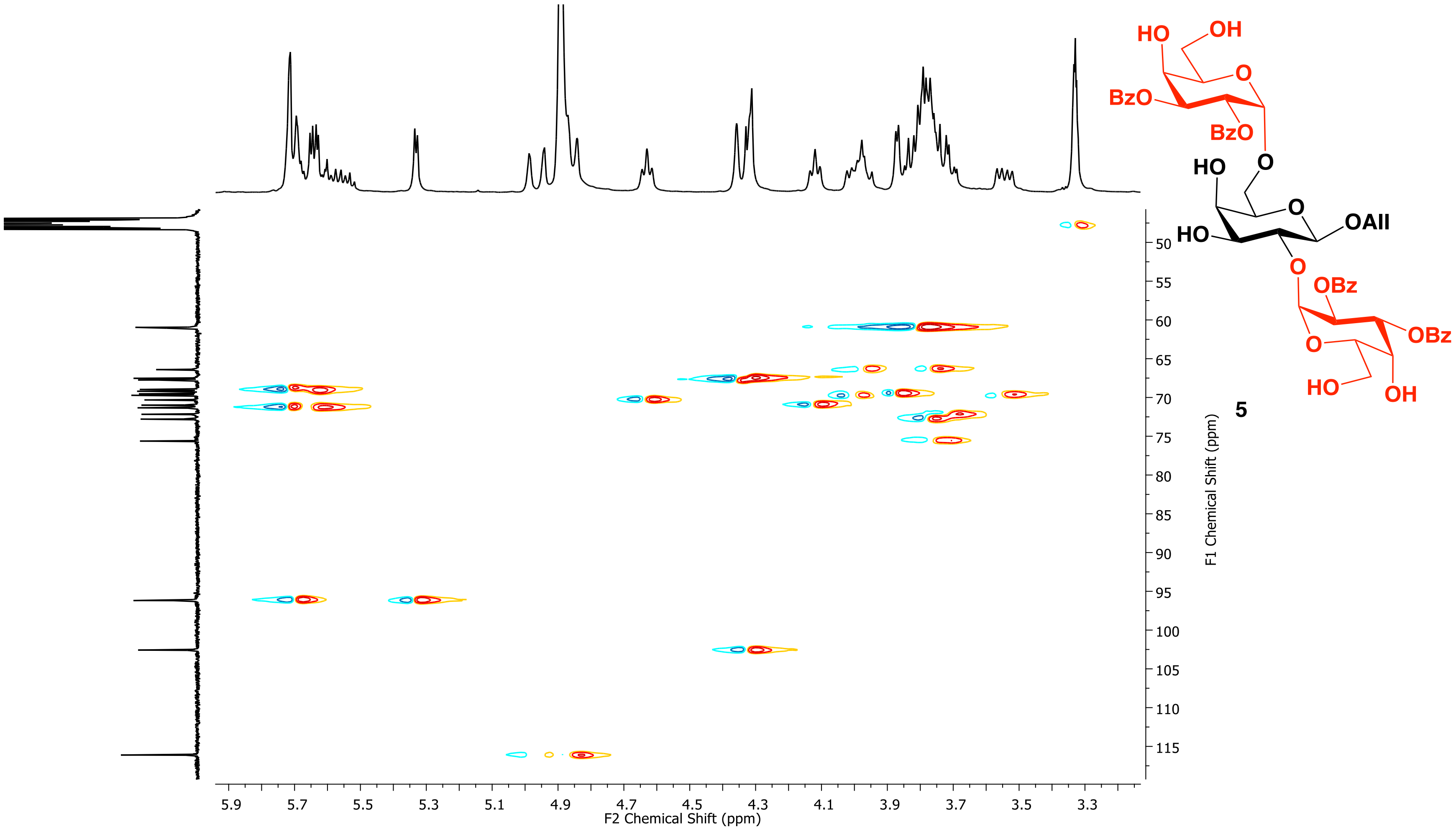

5

Figure S16: HSQC NMR spectrum of compound **5** (in methanol-d_4)

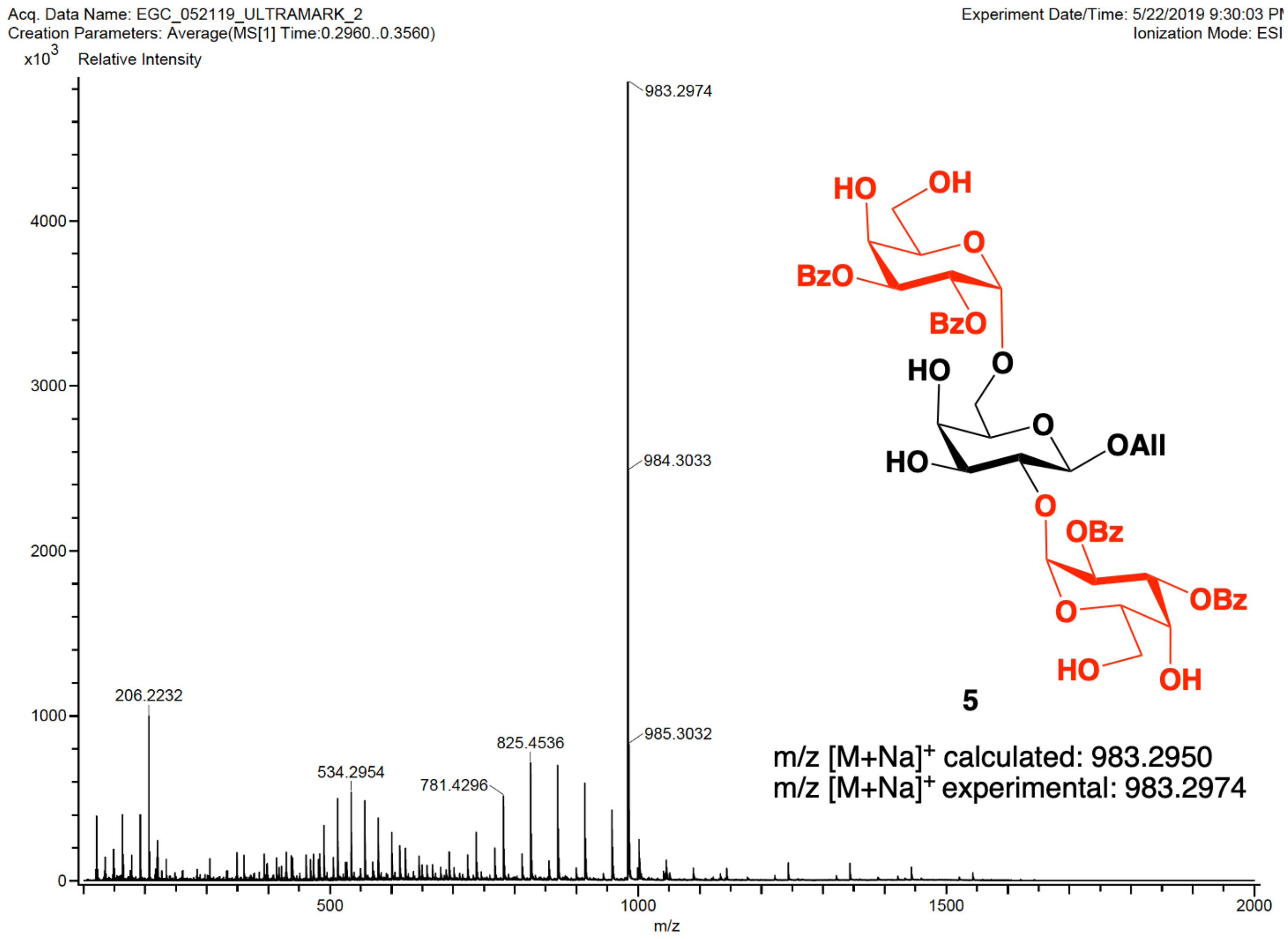

Figure S17: HR ESI-TOF mass spectrum of compound **5**

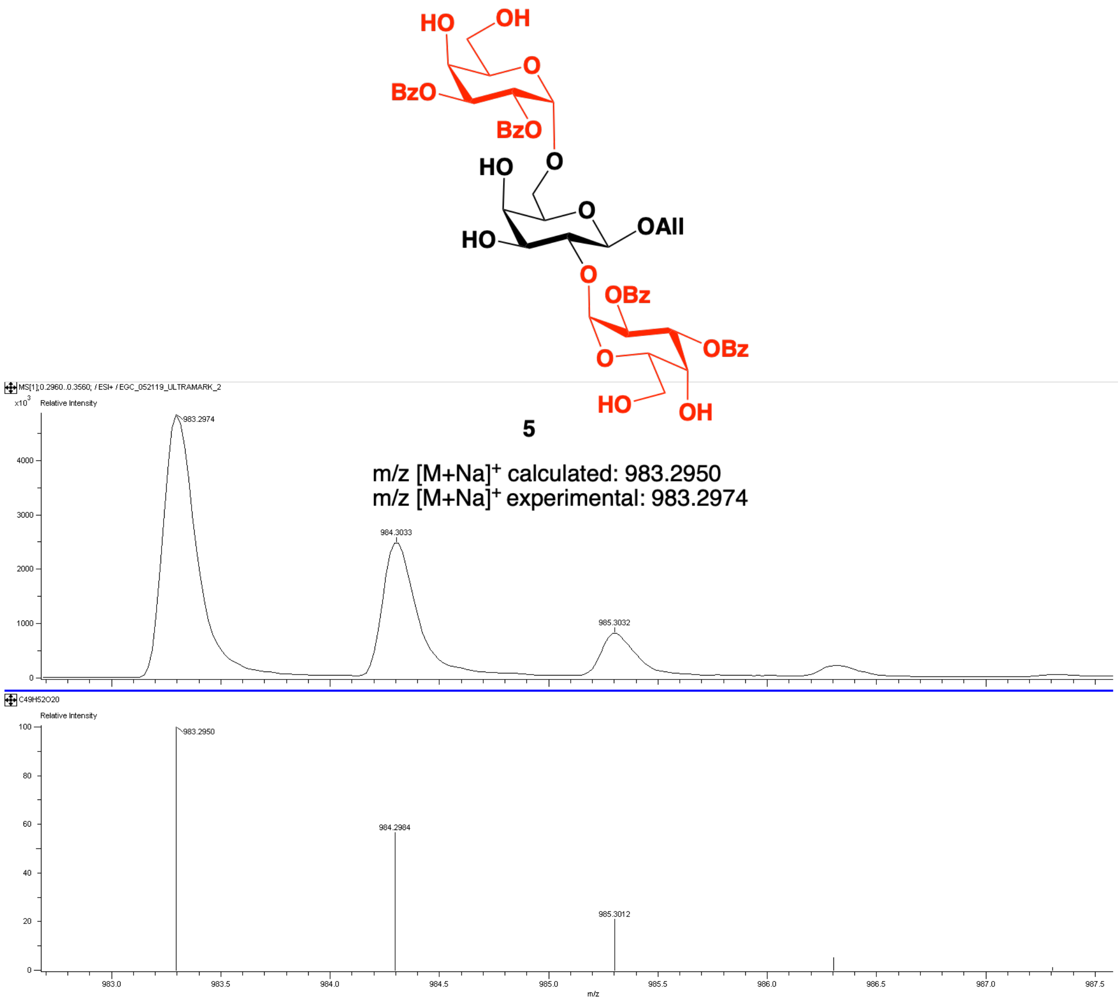

Figure S18: Actual (top) and simulated (bottom) HR ESI-TOF mass spectrum of compound **5**

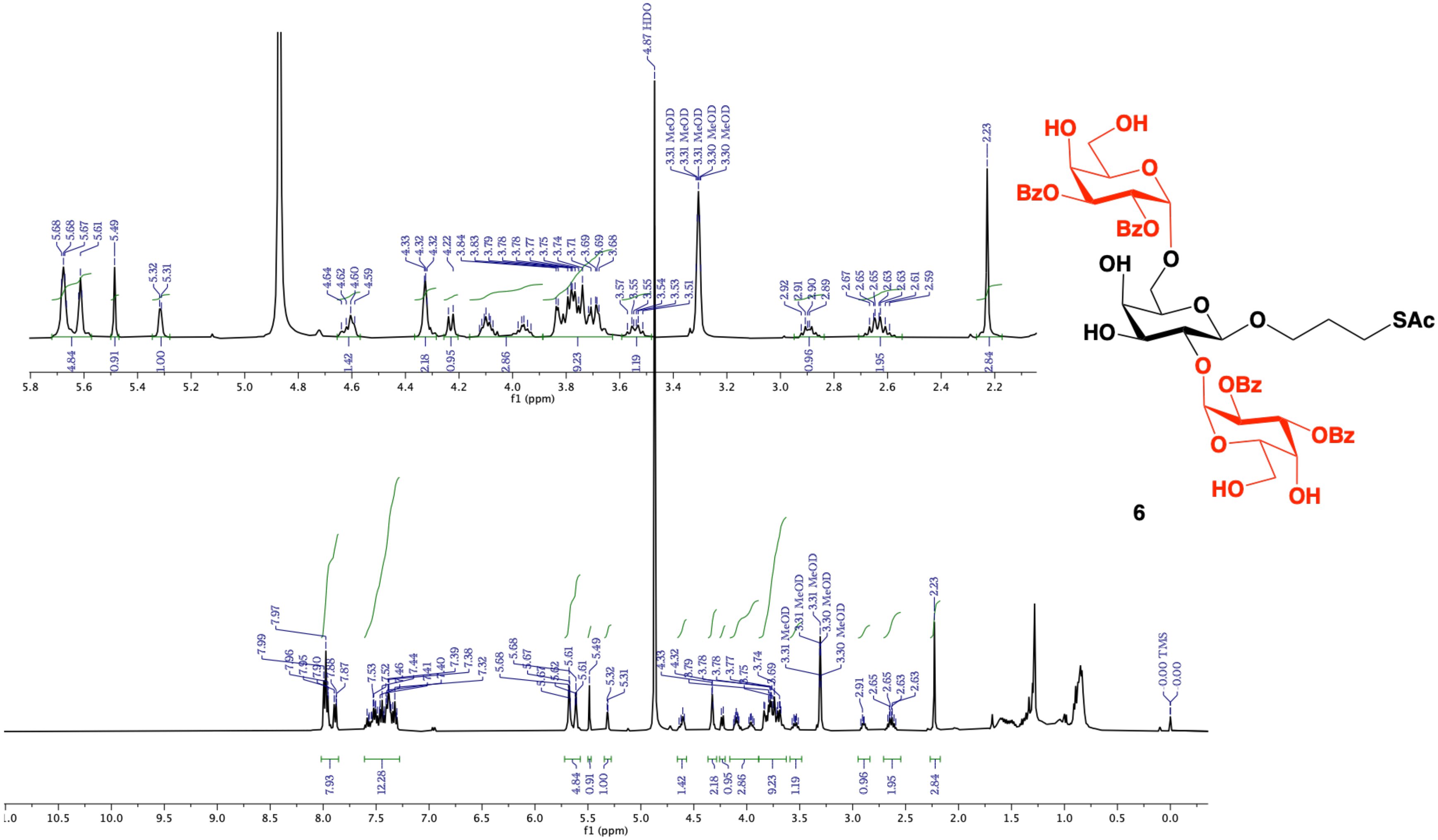

Figure S19: ^{1}H NMR spectrum of compound **6** (in methanol-d_4)

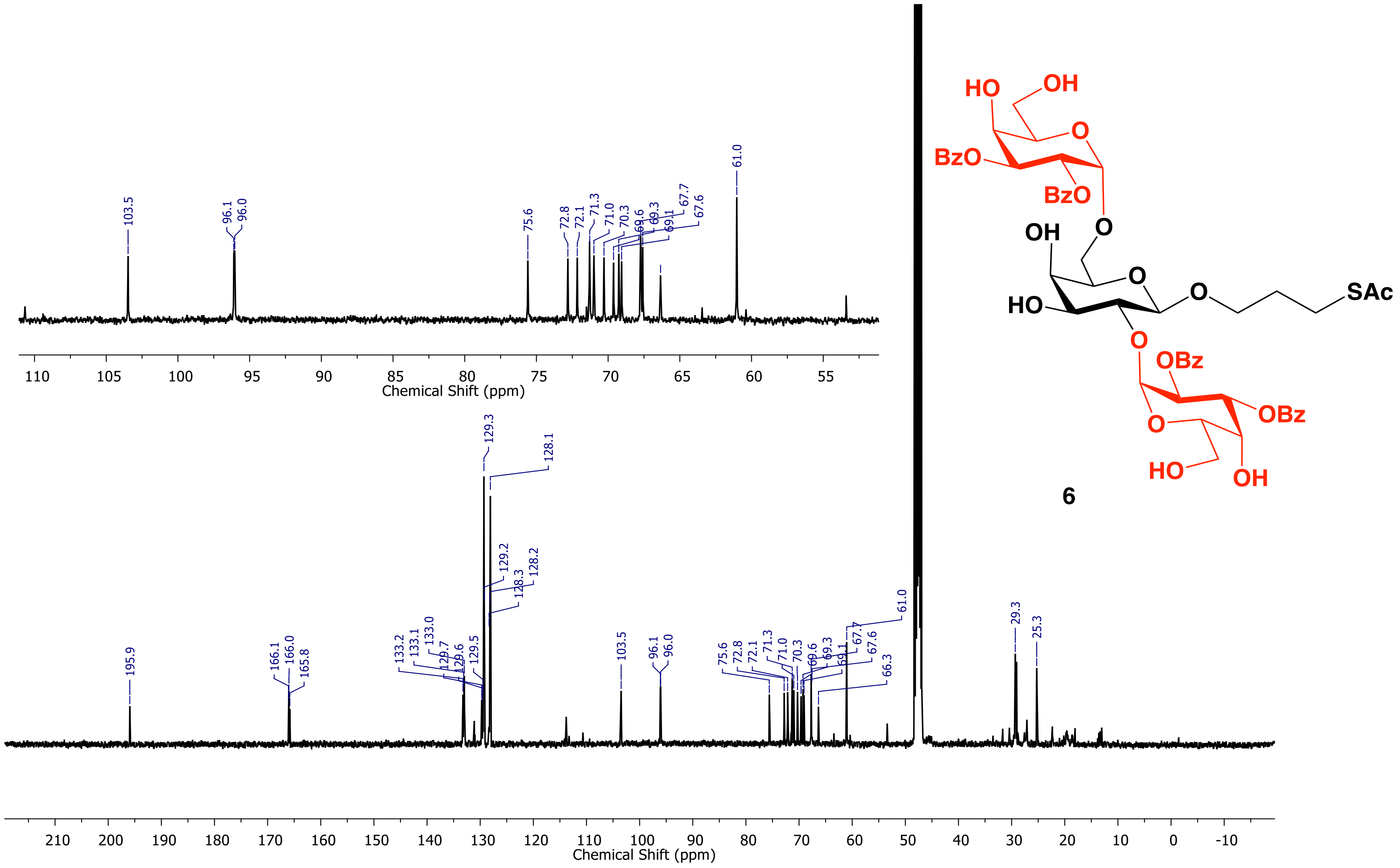

Figure S20: ^{13}C NMR spectrum of compound **6** (in methanol-d_4)

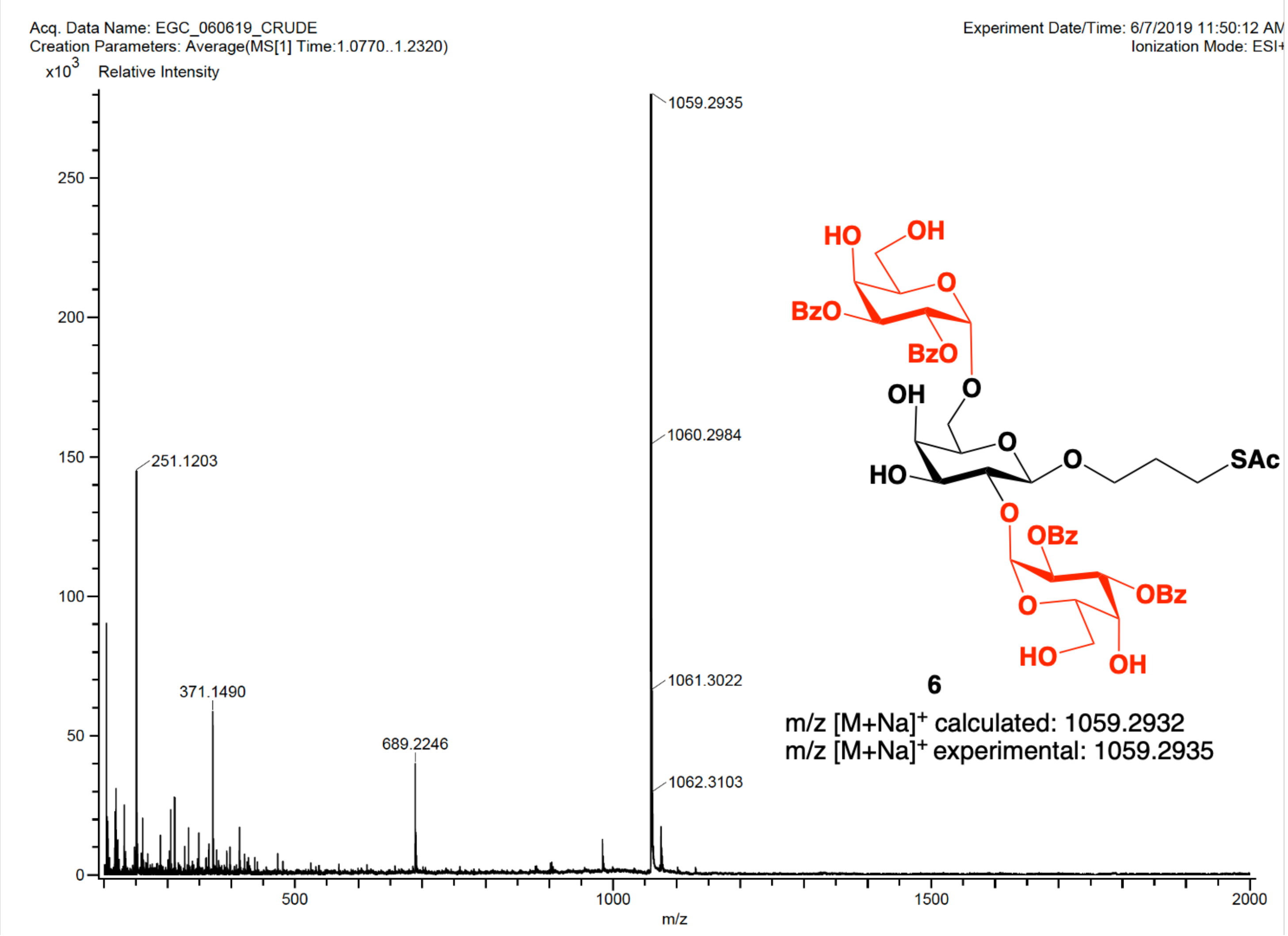

Figure S21: HR ESI-TOF mass spectrum of compound **6**

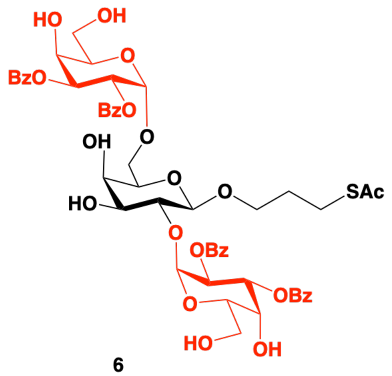

6

m/z $[M+Na]^+$ calculated: 1059.2932
m/z $[M+Na]^+$ experimental: 1059.2935

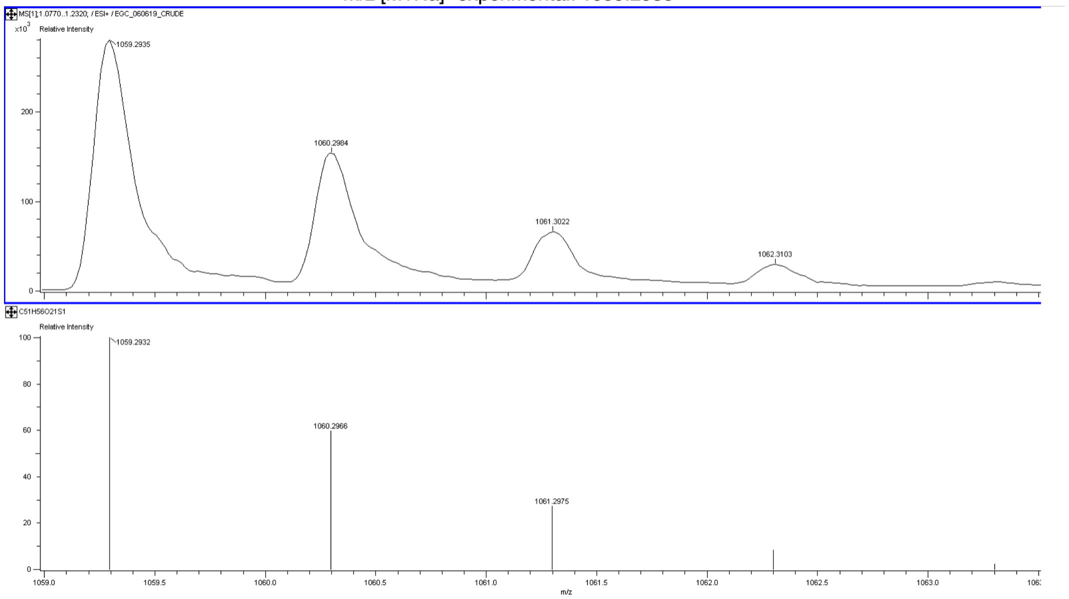

Figure S22: Actual (top) and simulated (bottom) HR ESI-TOF mass spectrum of compound **6**

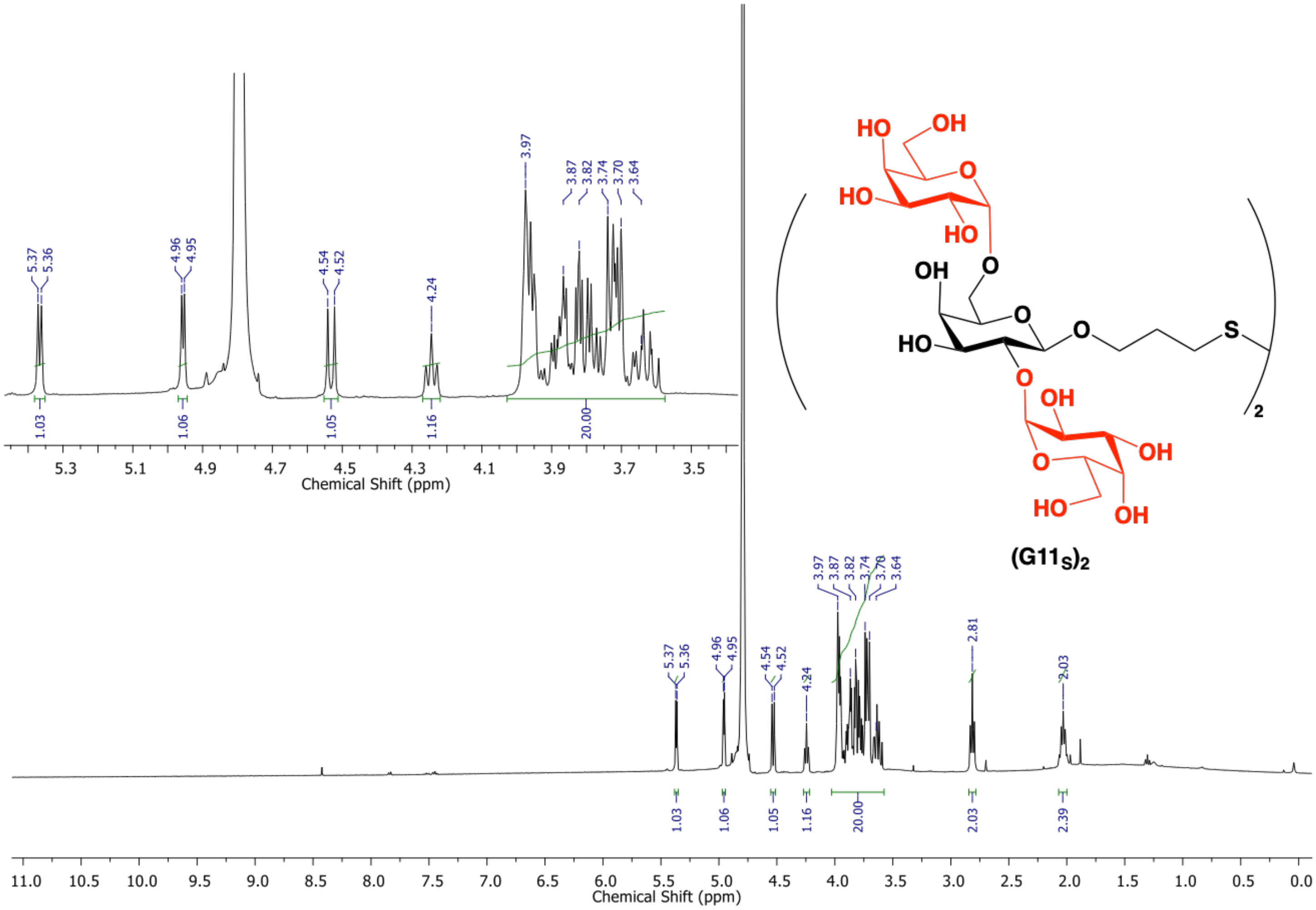

Figure S23: ^{1}H NMR spectrum of compound **$(G11_S)_2$** (400 MHz, in D_2O)

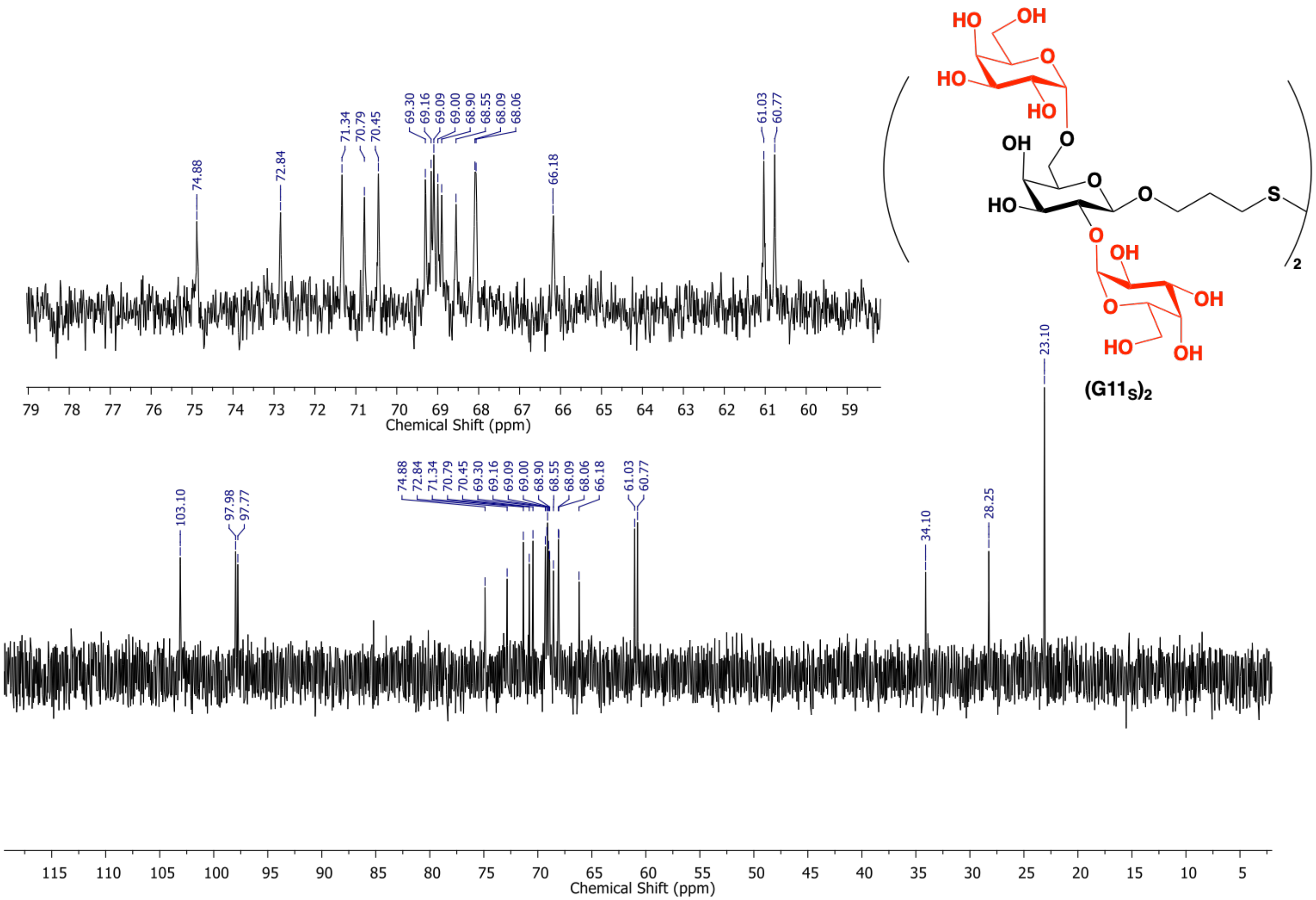

Figure S24: ^{13}C NMR spectrum of compound **$(G11_S)_2$** (101 MHz, in D_2O)

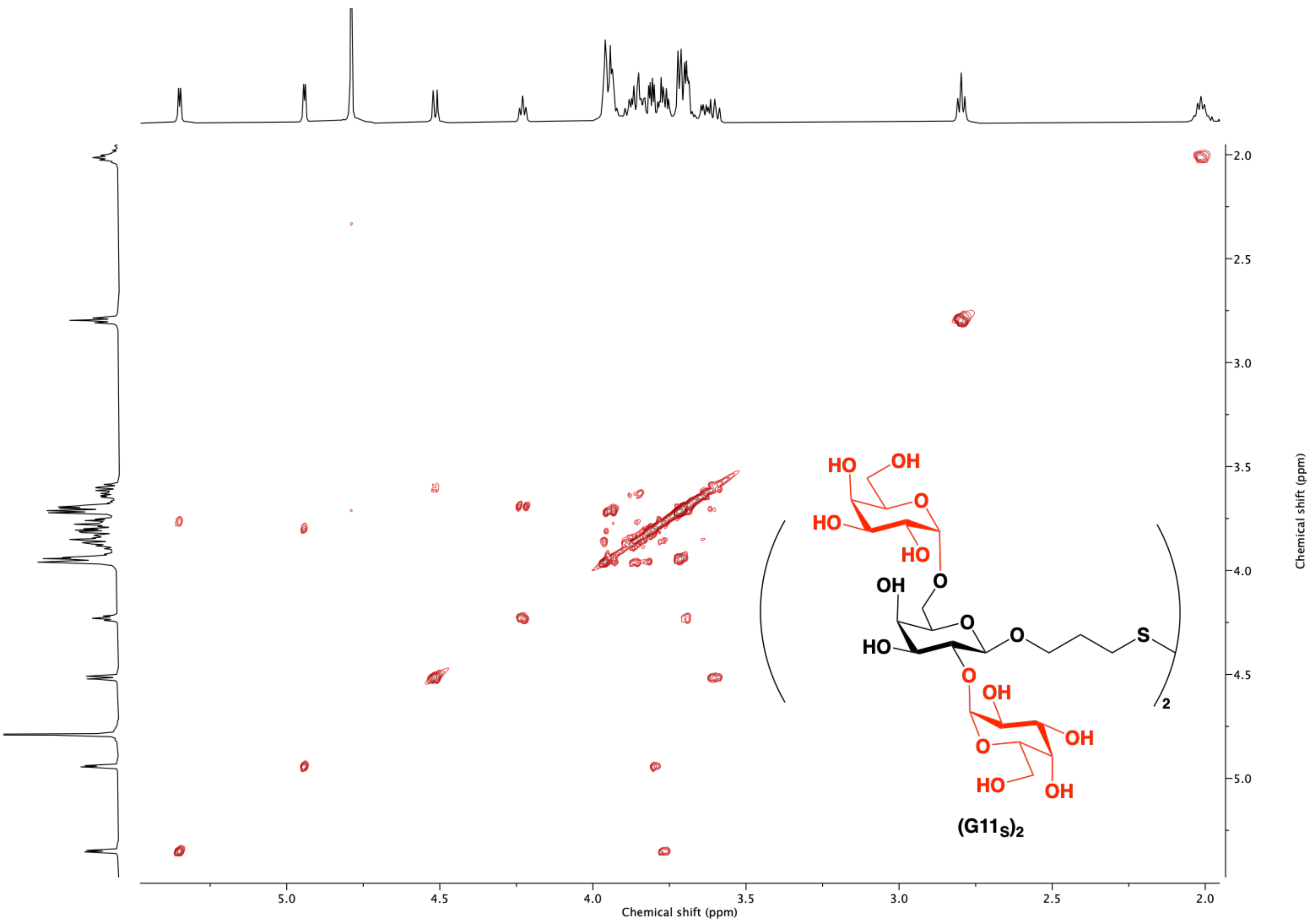

Figure S25: COSY NMR spectrum of compound **$(G11_S)_2$** (400 MHz, in D_2O)

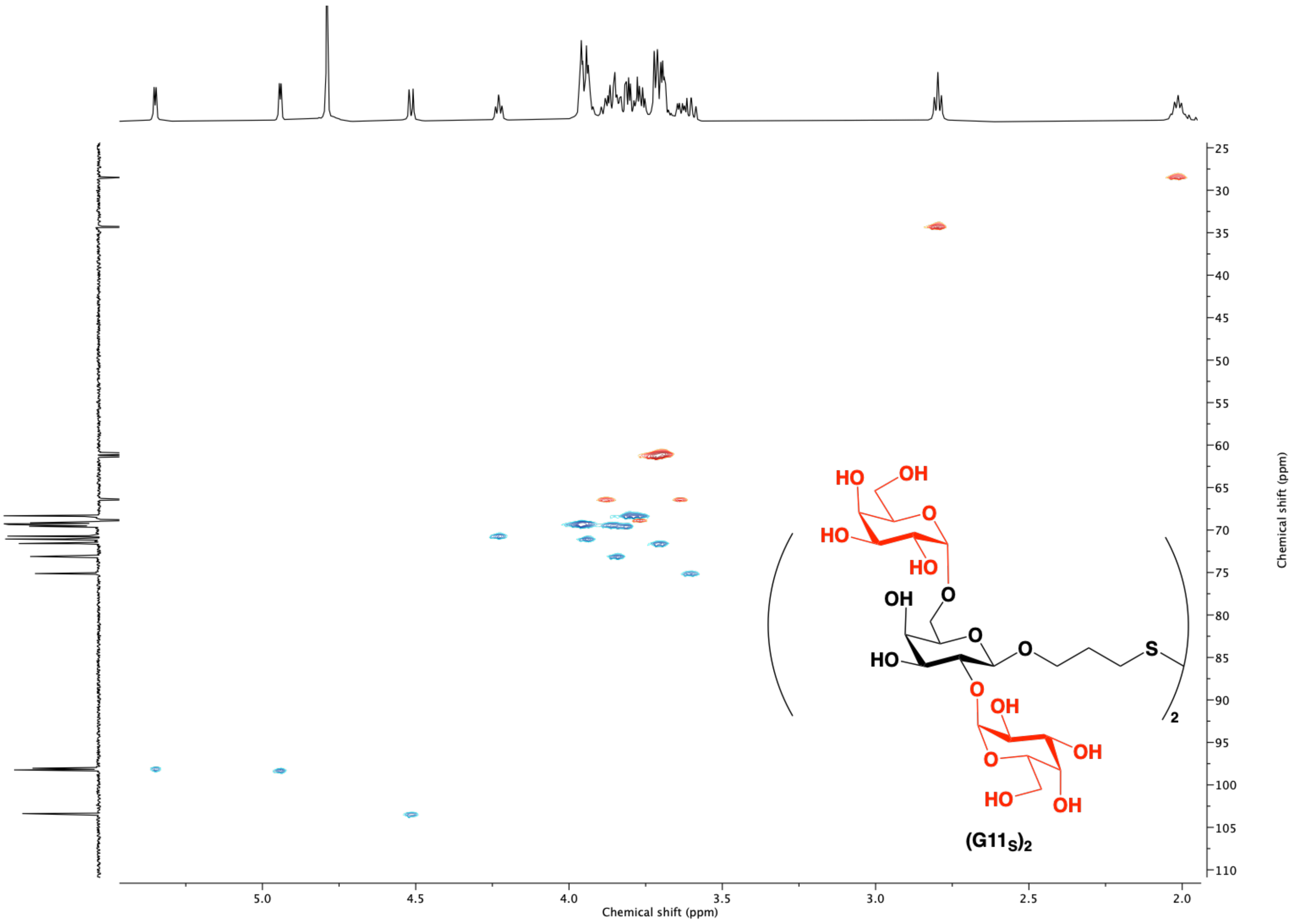

Figure S26: HSQC NMR spectrum of compound **(G11S)2** (in D_2O)

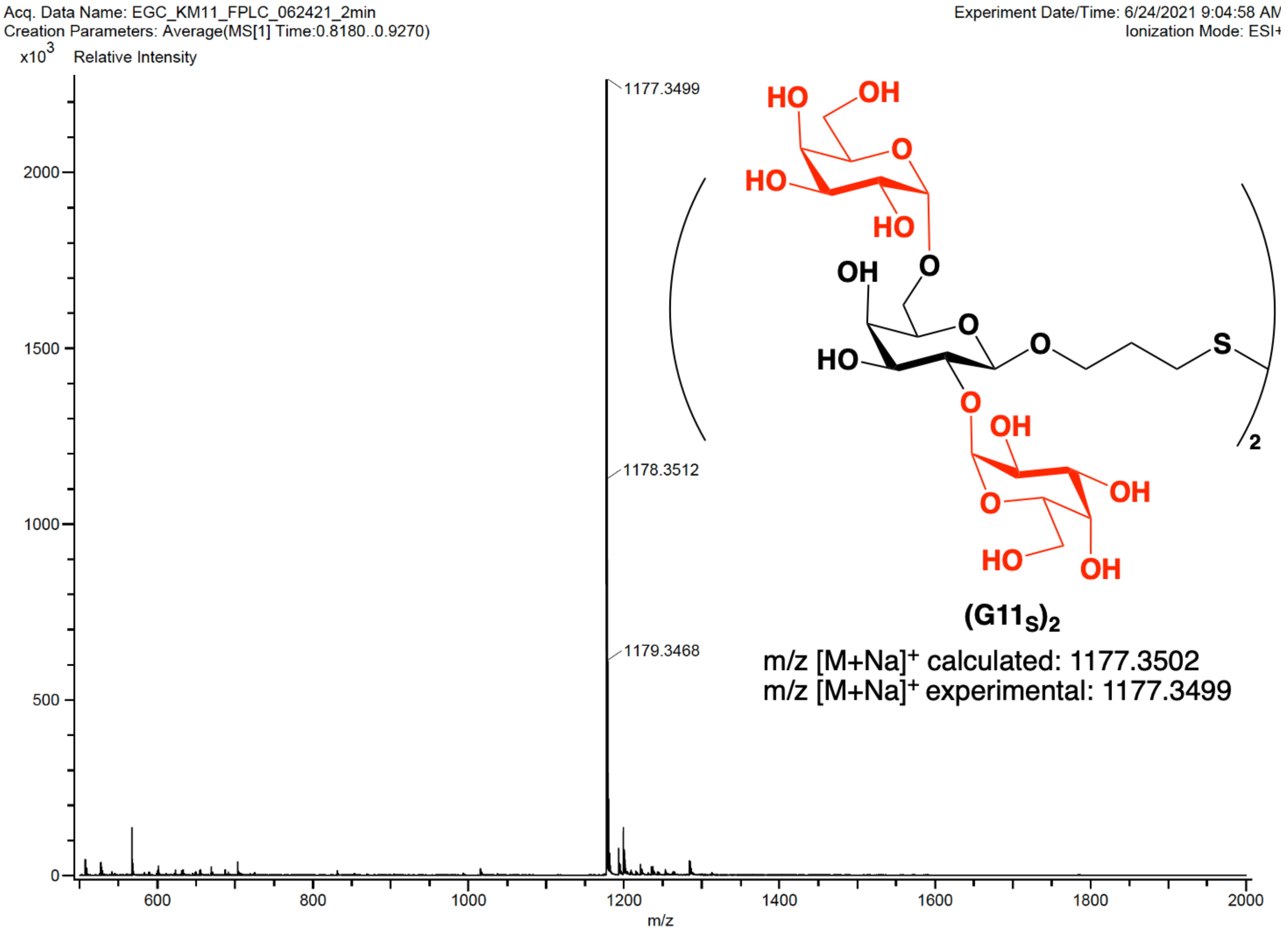

Figure S27: HR ESI-TOF mass spectrum of compound **$(G11_S)_2$**

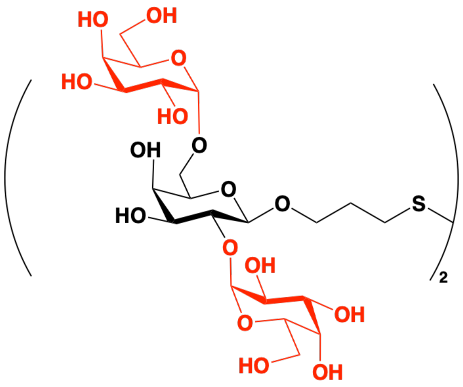

$(G11_S)_2$

m/z $[M+Na]^+$ calculated: 1177.3502
m/z $[M+Na]^+$ experimental: 1177.3499

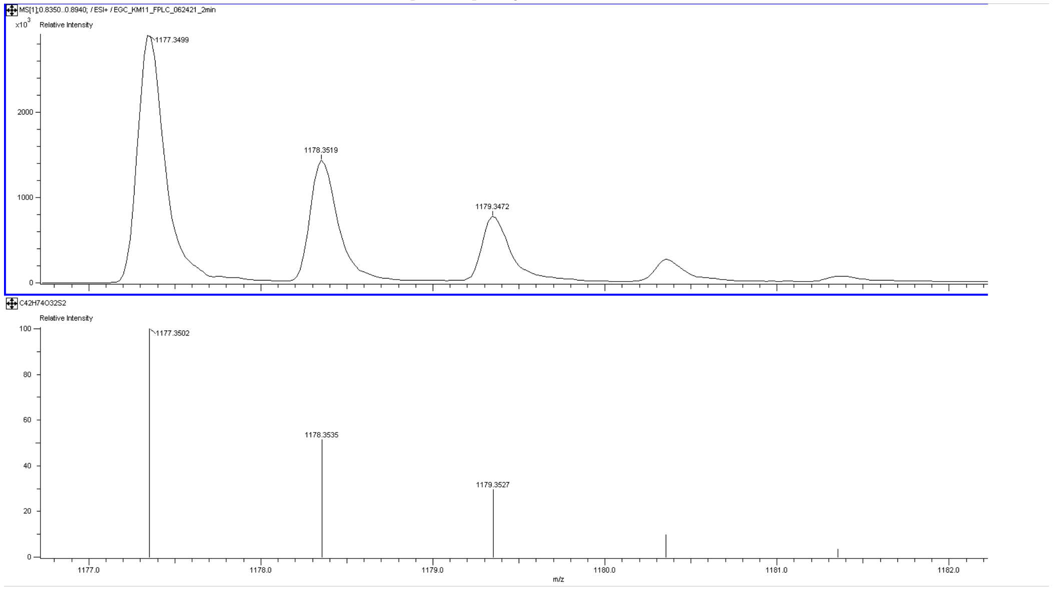

Figure S28: Actual (top) and simulated (bottom) HR ESI-TOF mass spectrum of compound **$(G11_S)_2$**
